# The Candidate Genes Underlying a Stably Expressed QTL for Low Temperature Germinability in Rice (*Oryza sativa* L.)

**DOI:** 10.1186/s12284-020-00434-z

**Published:** 2020-10-19

**Authors:** Tifeng Yang, Lian Zhou, Junliang Zhao, Jingfang Dong, Qing Liu, Hua Fu, Xingxue Mao, Wu Yang, Yamei Ma, Luo Chen, Jian Wang, Song Bai, Shaohong Zhang, Bin Liu

**Affiliations:** 1 Rice Research Institute, Guangdong Academy of Agricultural Sciences, Guangzhou, 510640 China; 2 Guangdong Key Laboratory of New Technology in Rice Breeding, Guangzhou, 510640 China

**Keywords:** Rice (*Oryza sativa* L.), Low temperature germinability, Quantitative trait locus, Candidate gene, Genome-wide association study

## Abstract

**Background:**

Direct seeding is an efficient cultivation technique in rice. However, poor low temperature germinability (LTG) of modern rice cultivars limits its application. Identifying the genes associated with LTG and performing molecular breeding is the fundamental way to address this issue. However, few LTG QTLs have been fine mapped and cloned so far.

**Results:**

In the present study, the LTG evaluation of 375 rice accessions selected from the Rice Diversity Panel 2 showed that there were large LTG variations within the population, and the LTG of *Indica* group was significantly higher than that of *Japonica* and *Aus* groups (*p* < 0.01). In total, eleven QTLs for LTG were identified through genome-wide association study (GWAS). Among them, *qLTG_sRDP2–3/qLTG_JAP-3*, *qLTG_AUS-3* and *qLTG_sRDP2–12* are first reported in the present study. The QTL on chromosome 10, *qLTG_sRDP2–10a* had the largest contribution to LTG variations in 375 rice accessions, and was further validated using single segment substitution line (SSSL). The presence of *qLTG_sRDP2–10a* could result in 59.8% increase in LTG under 15 °C low temperature. The expression analysis of the genes within *qLTG_sRDP2–10a* region indicated that *LOC_Os10g22520* and *LOC_Os10g22484* exhibited differential expression between the high and low LTG lines. Further sequence comparisons revealed that there were insertion and deletion sequence differences in the promoter and intron region of *LOC_Os10g22520*, and an about 6 kb variation at the 3′ end of *LOC_Os10g22484* between the high and low LTG lines, suggesting that the sequence variations of the two genes could be the cause for their differential expression in high and low LTG lines.

**Conclusion:**

Among the 11 QTLs identified in this study, *qLTG_sRDP2–10a* could also be detected in other three studies using different germplasm under different cold environments. Its large effect and stable expression make *qLTG_sRDP2–10a* particularly valuable in rice breeding. The two genes, *LOC_Os10g22484* and *LOC_Os10g22520*, were considered as the candidate genes underlying *qLTG_sRDP2–10a*. Our results suggest that integrating GWAS and SSSL can facilitate identification of QTL for complex traits in rice. The identification of *qLTG_sRDP2–10a* and its candidate genes provide a promising source for gene cloning of LTG and molecular breeding for LTG in rice.

## Background

Rice is the staple food for over half of the world population. Therefore, rice production plays a critical role in world food security. However, rice production is facing the threats of labor shortage, energy scarcity, decline of water table, and change in climatic conditions, which drive rice cultivation techniques to shift from traditional puddle transplanting to direct seeding (Singh et al. 2013). Being a simple, labor-saving and efficient cultivation technique, direct seeding has been adopted in many countries and will become an inevitable trend in rice-growing areas. No matter what environment, rapid and uniform germination during initial phase of seedling establishment is one of the essential phenotypic traits defined as early seedling vigour in rice direct seeding (Mahender et al. 2015). Due to tropical or subtropical origin, the optimum temperature for germination of cultivated rice is 25–35 °C, but the temperatures during sowing period are frequently below 15 °C in temperate and high elevation tropical and sub-tropical regions (Fujino et al. 2004). Poor germination and weak seedling establishment caused by low temperature, and subsequently decrease in yields has become one of the major factors limiting the application of direct seeding in rice.

Development and use of rice variety with high low temperature germinability (LTG) is the most economical way to solve this problem. However, the previous studies suggest that rice LTG is a quantitative trait controlled by multiple genes (Han et al. 2006; Jiang et al. 2006; Borjas et al. 2016; Satoh et al. 2016; Jiang et al. 2017). Therefore, it is difficult to develop rice variety with high LTG using the conventional breeding methods. Understanding the genetic basis of LTG is the key for high LTG rice breeding. The development and application of molecular marker technology and high through-put genotyping techniques provide powerful tools for genetic analysis of LTG in rice. So far, a large number of QTLs for LTG in rice have been identified using bi-parental QTL analysis (Miura et al. 2001; Fujino et al. 2004; Han et al. 2006; Jiang et al. 2006, 2017; Wang et al. 2011; Li et al. 2013; Ranawake et al. 2014; Borjas et al. 2016; Satoh et al. 2016). With the rapid development of genome sequencing technology, genome-wide association study (GWAS) based on linkage disequilibrium (LD) has emerged as a powerful tool for identifying the genes underlying complex traits in rice (Huang et al. 2010; McCouch et al. 2016). Compared with the bi-parental QTL analysis, GWAS is more efficient in identification of genes underlying complex traits due to use of a diverse natural population with more recombinant events and highly dense markers such as single nucleotide polymorphisms (SNPs). When population structure is reasonable and the molecular marker density is large enough, most of the loci associated with traits can be theoretically identified, and the accuracy of marker location can reach the level of gene (Yu and Buckler 2006). In the past few years, GWAS has been also used for identification of LTG QTLs in rice (Fujino et al. 2015; Sales et al. 2017; Schläppi et al. 2017; Shakiba et al. 2017; Wang et al. 2018a, b), and some loci overlap with the previous LTG QTLs identified by bi-parental QTL analysis.

Progress has been made in fine mapping and cloning of LTG QTLs in rice. The *qLTG3–1* has been successfully cloned using map-based cloning approach (Fujino et al. 2008). The *qLTG3–1* is strongly expressed in the embryo during seed germination and tightly associated with vacuolation, and the elevated vacuolation observed in the NIL suggests that the function of *qLTG3–1* may be to accelerate vacuolation and to be involved in the weakening of tissues covering the embryo during the seed germination process (Fujino et al. 2008). The major-effect LTG QTL, *qLTG-9* has been fine mapped to a 72.3 kb region on chromosome 9 by using near-isogenic lines and five candidate genes were identified (Li et al. 2013). In addition, Wang et al. (2018a) performed a GWAS of LTG using 187 rice accessions under 12 °C low temperature and confirmed that *OsSAP16* (Stress-Associated Protein 16, *LOC_Os07g38240*) coding a zinc-finger domain protein was the causal gene of QTL *qLVG7–2*. Loss of *OsSAP16* function reduces germination while greater expression of *OsSAP16* enhances germination at low temperature. The rice accessions with extremely high and low LTG exhibited correspondingly high and low *OsSAP16* expression levels under low temperatures, suggesting that the variation in expression of the *OsSAP16* contributes to LTG variation in the natural accessions tested.

Although numerous LTG QTLs in rice have been identified and mapped, these QTLs have not been effectively used in rice breeding. The uncertainty of these QTLs could be one of the main reasons related to this issue. The identified QTLs should be validated, fine mapped or even cloned before marker-assisted selection can be effectively deployed. However, most of the identified LTG QTLs in rice have not been validated and few of them have been fine mapped and cloned. On the other hand, rice LTG is a quantitative trait controlled by multiple genes and the expression of QTL for LTG is frequently affected by environments and genetic backgrounds. Thus, identifying the LTG QTL with large effect and stable expression in different cold environments and genetic backgrounds will be pivotal for LTG rice breeding. Furthermore, plant height is one of the important traits which should be considered in direct-seeding cultivation because tall rice plants are susceptible to lodging and resulting in decrease in yields. The previous studies reported that gibberellins (GAs) play important roles in seed germination, stem elongation, and flower development (Yamaguchi and Kamiya 2000; Olszewski et al. 2002). Recent studies demonstrated that genes involving in GA synthesis, *OsGA20ox1*, *OsGA20ox2* and *OsKO1* promotes germination and enhances plant height in rice (Abe et al. 2012; Ye et al. 2015; Zhang et al. 2020), while QTLs for seedling height and seed dormancy co-located with *OsGA20ox1* (Abe et al. 2012) and *OsGA20ox2* (Ye et al. 2015), respectively. Therefore, it could be possible that some LTG QTLs enhance seed germination under low temperature, and also contribute to increase in plant height, such QTLs could not be used in rice breeding for direct-seeding due to the risk of lodging.

In this study, we conducted GWAS of LTG using a subset of the Rice Diversity Panel 2 (sRDP2) consisting of 375 rice accessions, which has been genotyped by 700 K SNPs (McCouch et al. 2016). In total, eleven LTG QTLs were identified and located on chromosomes 1, 3, 4, 5, 7, 10 and 12. Among them, *qLTG_sRDP2–10a* on chromosome 10 had the largest contribution to LTG variations in 375 rice accessions. This QTL was further validated using single segment substitution line (SSSL) and its presence resulted in 59.8% increase in LTG under low temperature. It is noteworthy that three previous studies also mapped QTLs for LTG to the same regions as *qLTG_sRDP2–10a* (Jiang et al. 2006; Wang et al. 2018a, b). Through integrating differential expression and sequence variation analysis of the genes within the region of *qLTG_sRDP2–10a* between the rice accessions with high and low LTG, two candidate genes were identified. Since *qLTG_sRDP2–10a* was detected in five studies using different rice germplasm under different low temperatures, it could be a stably expressed QTL for LTG. The identification of *qLTG_sRDP2–10a* and its candidate genes provide a promising source for functional gene identification of LTG and molecular breeding for LTG in rice.

## Results

### Variations of Low Temperature Germinability in sRDP2 Population

Phylogenetic analysis based on their genotypes determined by the 700 K SNPs (McCouch et al. 2016) demonstrated that the 375 rice accessions could be clustered into three groups, representing *Indica* (154 accessions), *Japonica* (147 accessions) and *Aus* (74 accessions) (Fig. S1A).

**Fig. 1:**
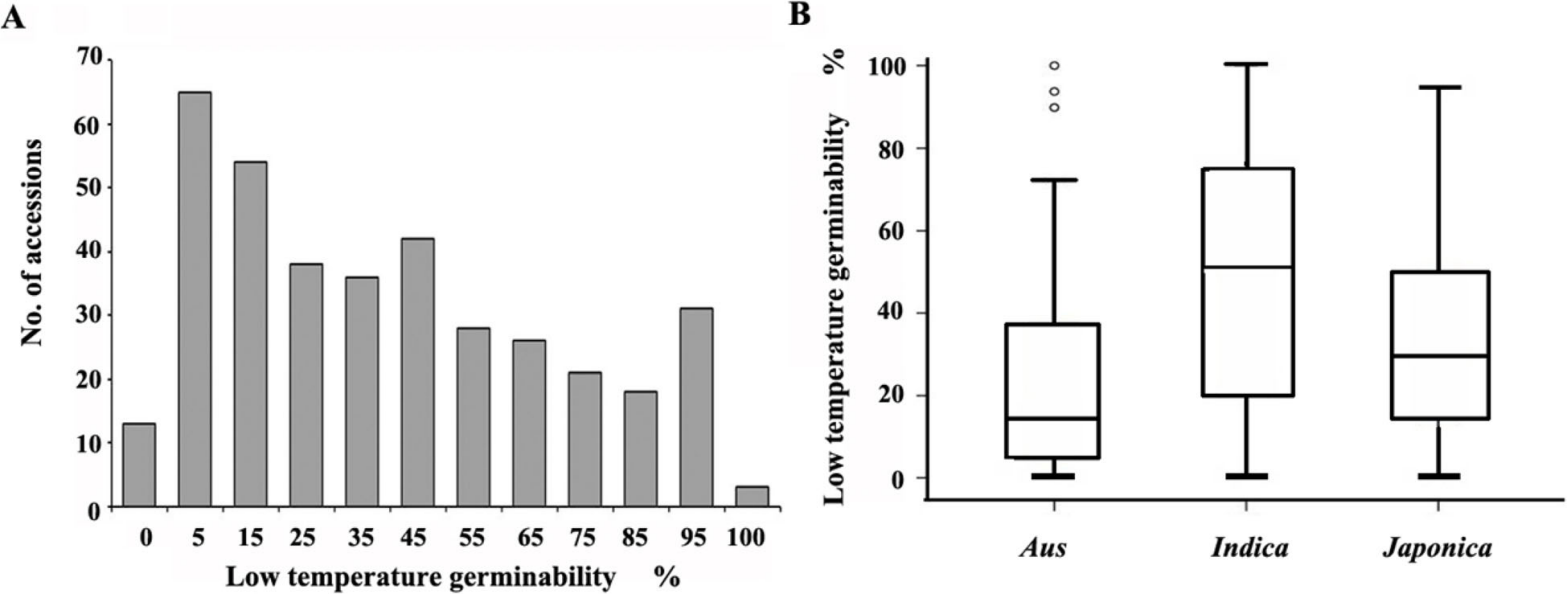
Distribution and variations of low temperature germinability in the 375 rice accessions. **a** Distribution of LTG in 375 rice accessions. **b** Boxplot of the LTG variation in the three subpopulations. The black horizontal lines represent the median value; The upper side and lower side of the box represent the upper quartile and lower quartile, respectively; The whiskers represent the range of data, and small circle represents outlier

Large variations in LTG were observed in sRDP2 population under 13 °C low temperature (Table S1), ranging from 0 to 100.0%, with an average of 39.2% and a variation coefficient of 75.7%. The LTG distribution in 375 accessions was continuous, with more in the low LTG side (Fig. 1a). Seventy-eight accessions (20.8%) had LTG less than 10.0%, while 34 accessions (9.1%) had LTG more than 90.0% (Table S2). Particularly, the three accessions, DJOGOLON (accession 491, *Indica*), HONG ZUI ER (accession 517, *Indica*) and KHADASIYA 3 (accession 902, *Aus*) had LTG of 100%.

The LTG comparisons among different groups revealed that the LTG of *Indica* group was significantly higher than that of *Japonica* and *Aus* groups (*p* < 0.01), while the LTG of *Japonica* group was significantly higher than that of *Aus* group (*p* < 0.05) (Fig. 1b).

### Identification and Mapping of QTLs for LTG Through GWAS

**Fig. 2:**
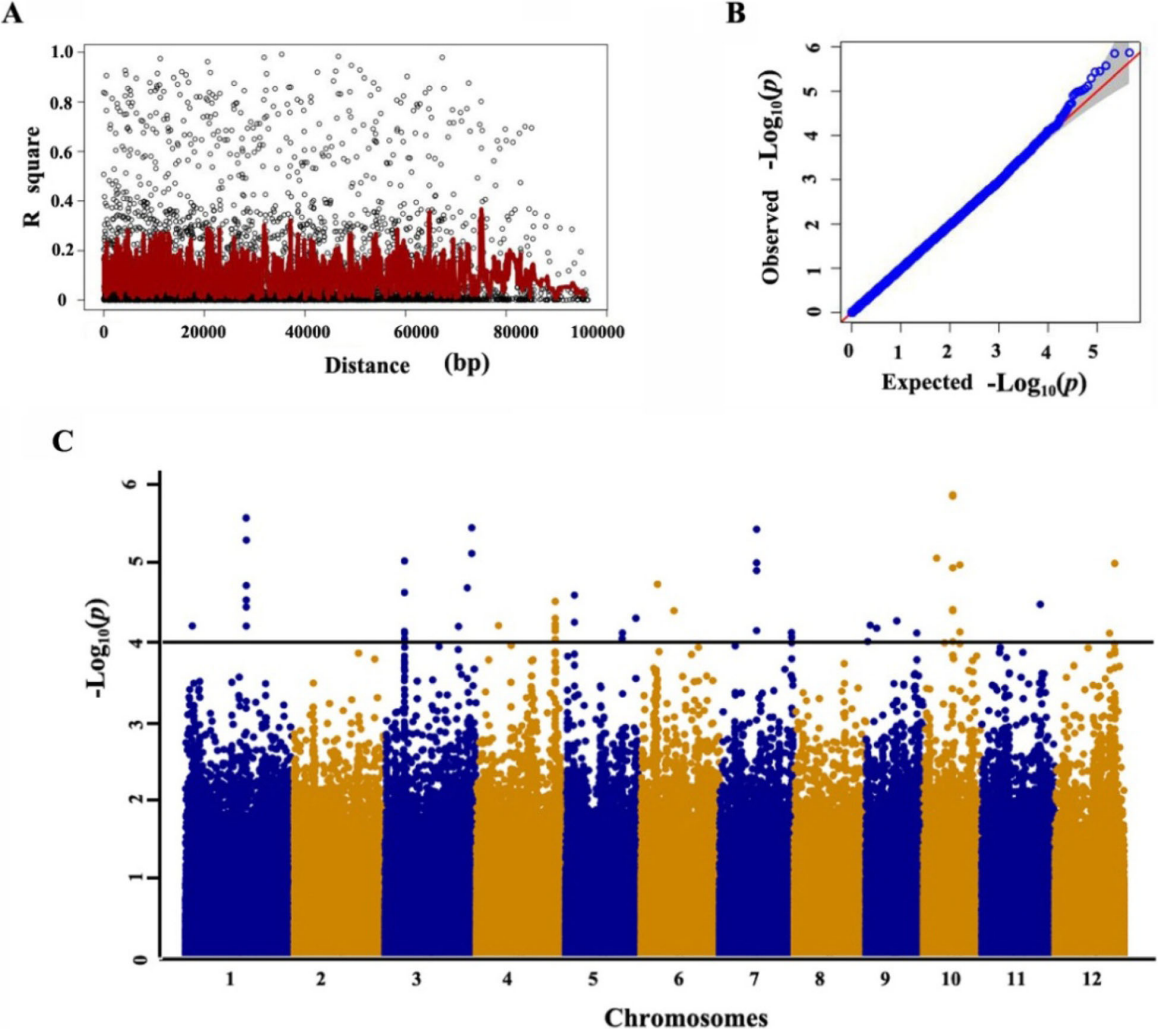
Genome-wide association study of low temperature germinability in 375 rice accessions. **a** LD decay in 375 rice accessions. **b** Quantile-quantile (Q-Q) plot of *p*-values for LTG. **c** Manhattan plots of GWAS for low temperature germinability in 12 chromosomes

Based on the criteria of having less than 30% missing data and minor allele frequency (MAF) more than 5% in the sRDP2 population, 445,534 SNPs were selected for GWAS from the 700 K SNPs dataset in the Open Rice GWAS Platform (McCouch et al. 2016). Population structure of the sRDP2 estimated by Admixture software, principal component analysis (PCA) and kinship analysis suggested that there were three subpopulations in this panel, which are consistent to *Indica*, *Japonica* and *Aus* subpopulation (Fig. S1). Considering the effect of population structure on GWAS, three PCs were included in the mixed linear model (MLM) with kinship matrix when analyzing the whole population. The QQ plot and Manhattan plot of the GWAS results were showed in Fig. 2b and c. According to about 100 kb of the LD decay in 375 rice accessions (Fig. 2a), we delimited a QTL to a 200-kb region with center on the most significant SNP, in which there are three or more than three significant SNPs (*p* < 0.0001). Accordingly, nine QTLs with 35 significant SNPs were identified in the whole population (Fig. 2c). These QTLs distributed on chromosomes 1, 3, 4, 5, 7, 10, 12 and were designated as *qLTG_sRDP2–1*, *qLTG_sRDP2–3*, *qLTG_sRDP2–4*, *qLTG_sRDP2–5*, *qLTG_sRDP2-7a*, *qLTG_sRDP2-7b*, *qLTG_sRDP2–10a*, *qLTG_sRDP2–10b* and *qLTG_sRDP2–12*. Among the 9 QTLs, *qLTG_sRDP2–3* and *qLTG_sRDP2–12* located on chromosomes 3 and 12, respectively, are first reported in the present study, and the other seven QTLs co-localized with the previously identified LTG QTLs (Table 1). The QTL, *qLTG_sRDP2–10a* with the most significant SNP at 11,648,047 bp in the region of 11.55–11.75 Mb on chromosome 10 had the largest contribution to LTG variation in 375 rice accessions. It is noteworthy that this LTG QTL was also detected in the previous three studies using different rice germplasm under different low temperatures (Jiang et al. 2006; Wang et al. 2018a, b) (Table 1), suggesting that it is a stably expressed QTL for LTG.

**Table 1:** QTLs for low temperature germinability identified in the present study and their co-location cold tolerant QTLs identified in the previous studies

QTL	Chromosome	The most significant SNP position (bp)^a^	*P*-value	Variation explained (%)	Co-location QTL^b^	Reference
**Whole population**						
*qLTG_sRDP2–1*	1	24,086,631	2.68E-06	4.74	SNP-1.23843134^c^	Wang et al. 2018a
					*qLTSSvR1–2*	Pan et al. 2015
*qLTG_sRDP2–3*	3	7,834,390	9.43E-06	4.21		
*qLTG_sRDP2–4*	4	31,340,881	3.10E-05	3.71	*qLTG-4*^c^	Jiang et al. 2006
					*qCTGERM4–5*^c^	Shakiba et al. 2017
					*Ctb1*	Saito et al. 2010
*qLTG_sRDP2–5*	5	3,504,648	2.58E-05	3.79	*qLTG-5-1*^c^	Jiang et al. 2006
*qLTG_sRDP-7a*	7	14,730,833	3.72E-06	4.60	*qCTG-7-2*^c^	Ranawake et al. 2014
*qLTG_sRDP2-7b*	7	28,597,440	7.72E-05	3.33	*qLTSSvR7–2*	Pan et al. 2015
					*qLTG-7*^c^	Schläppi et al. 2017
					SNP-7.28369125^c^	Wang et al. 2018a
*qLTG_sRDP2–10a*	10	11,648,047	1.36E-06	5.03	*qLTSS10–2*	Schläppi et al. 2017
					SNP-10.11494085^c^	Wang et al. 2018a
					chr10_11890928^c^	Wang et al. 2018b
					*qLTG-10*^c^	Jiang et al. 2006
					*qCTSR10–1*	Zhang et al. 2018
*qLTG_sRDP2–10b*	10	14,457,620	1.06E-05	4.16	*qLTG-10*^c^	Jiang et al. 2006
*qLTG_sRDP2–12*	12	23,790,019	1.02E-05	4.18	*qLTSS-12*	Schläppi et al. 2017
**Japonica**						
*qLTG_JAP-3*	3	7,769,981	8.47E-06	13.23		
**Aus**						
*qLTG_AUS-3*	3	779,807	9.02E-06	24.02		
*qLTG_AUS-4*	4	31,506,890	4.99E-05	19.58	*qLTG-4*^c^	Jiang et al. 2006
					*qCTGERM4–5*^c^	Shakiba et al. 2017
					*Ctb1*	Saito et al. 2010
*qLTG_AUS-7*	7	20,735,661	1.72E-06	28.55	*qCTGERM7–4*^c^	Shakiba et al. 2017

^a^Position of the most significant SNP at the QTL region

^b^Cold tolerance QTLs identified by the previous studies

^c^Indicates the QTL for low temperature germinability

**Fig. 3:**
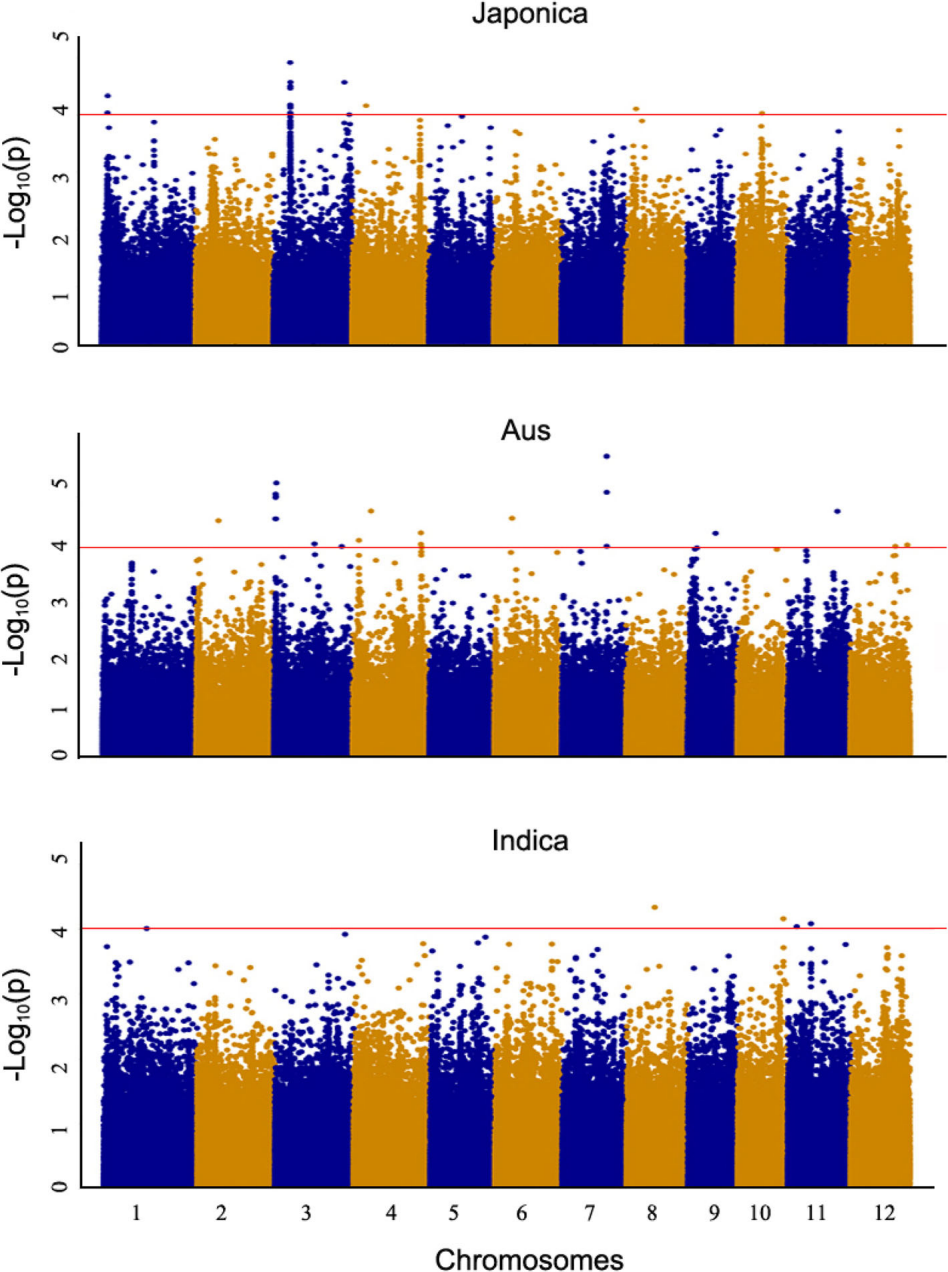
Manhattan plots of GWAS for low temperature germinability using subpopulations

GWAS was further conducted on the *Indica*, *Aus* and *Japonica* subpopulations and compared with all the identified QTLs (Fig. 3). In total, three and one QTLs were identified in the *Aus* and *Japonica* subpopulations, respectively, but none was identified in *Indica* subpopulation based on the criterion having more than three SNPs with the *p* < 0.0001 within 200-kb region. Comparisons of the QTLs identified in different subpopulations indicated that *qLTG_JAP-3* from the *Japonica* subpopulation and *qLTG_AUS-4* from the *Aus* subpopulation overlapped with *qLTG_sRDP2–3* and *qLTG_sRDP2–4* identified in the whole population, respectively, while *qLTG_AUS-3* and *qLTG_AUS-7* were only identified in the *Aus* subpopulation. *qLTG_AUS-3* is also first reported in the present study (Fig. 3 and Table 1).

### Validation of *qLTG_sRDP2–10a* Using Single Segment Substitution Line

**Fig. 4:**
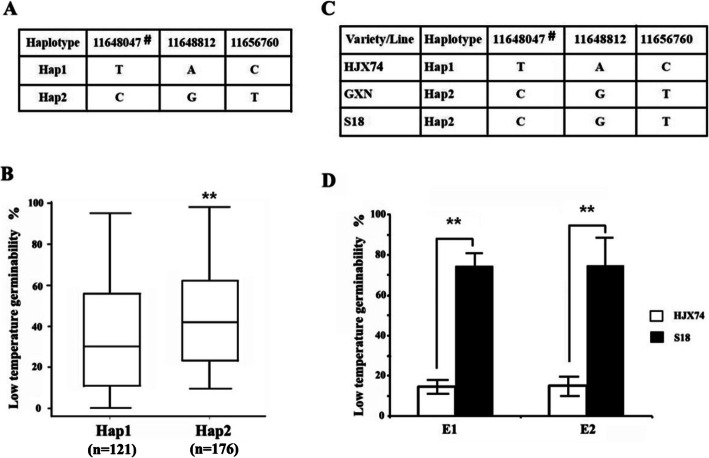
Validation of *qLTG_sRDP2–10a* using single segment substitution line. **a** The haplotypes of *qLTG_sRDP2–10a*, ^#^ The SNP position (bp). **b** Boxplots for low temperature germinability based on the haplotypes (Hap1 and Hap2) of *qLTG_sRDP2–10a* in the 297 rice accessions. n represents the number of accessions with the haplotype. **c** Haplotypes of SSSL (S18), and its donor parent (GXN) and recurrent parent (HJX74) in *qLTG_sRDP2–10a* region. ^#^ The SNP position (bp). **d** Comparisons of low temperature germinability between HJX74 and S18 in two independent experiments. E1 and E2 represent two independent experiments, respectively. **indicates the significant difference at *p* < 0.01 based on *t-*test

Being stably expressed in different genetic background and cold environments, it is believed that *qLTG_sRDP2–10a* has a great potential value in rice breeding for LTG. To search for single segment substitution lines (SSSLs) for validation of *qLTG_sRDP2–10a*, haplotype analysis of *qLTG_sRDP2–10a* was performed and the haplotypes associated with high and low LTG were identified (Fig. 4a). The accessions with haplotype 2 (Hap2) had a LTG significantly higher than that with haplotype 1 (Hap1) (*p <* 0.01) (Fig. 4b). Based on the two haplotypes, we screened the rice SSSLs bank developed by Zhang et al. (2004) and identified Gangxiangnuo (GXN, donor parent carrying Hap2), Huajingxian74 (HJX74, recurrent parent carrying Hap1) and their derived SSSL S18 with a substitution segment in 10.99–11.96 Mb on chromosome 10 from GXN (Fig. 4c). The high LTG haplotype (Hap2) was found in GXN and S18, indicating that *qLTG_sRDP2–10a* exists in GXN and S18. Our previous study indicated that the two parents GXN and HJX74 exhibited significant difference in LTG, with 85.3% and 20.5% under 15 °C low temperature, respectively (Yang et al. 2017). In this study, the LTG assessments in two independent experiments showed that the LTG of S18 was 74.6% and 74.8%, respectively, also significantly higher than that of HJX74 with respective LTG 14.7% and 15.1% under 15 °C low temperature (*p* < 0.01) (Fig. 4d). Thus, *qLTG_sRDP2–10a* is validated and its presence could result in 59.8% increase in LTG under 15 °C low temperature. Further studies indicated that there is no significant difference in plant height between HJX74 and S18 (*p >* 0.05), with 112.0 cm and 112.5 cm, respectively (Fig. S2), suggesting that the introgression of *qLTG_sRDP2–10a* had no effect on the plant height.

### Candidate Gene Analyses of *qLTG_sRDP2–10a*

**Fig. 5:**
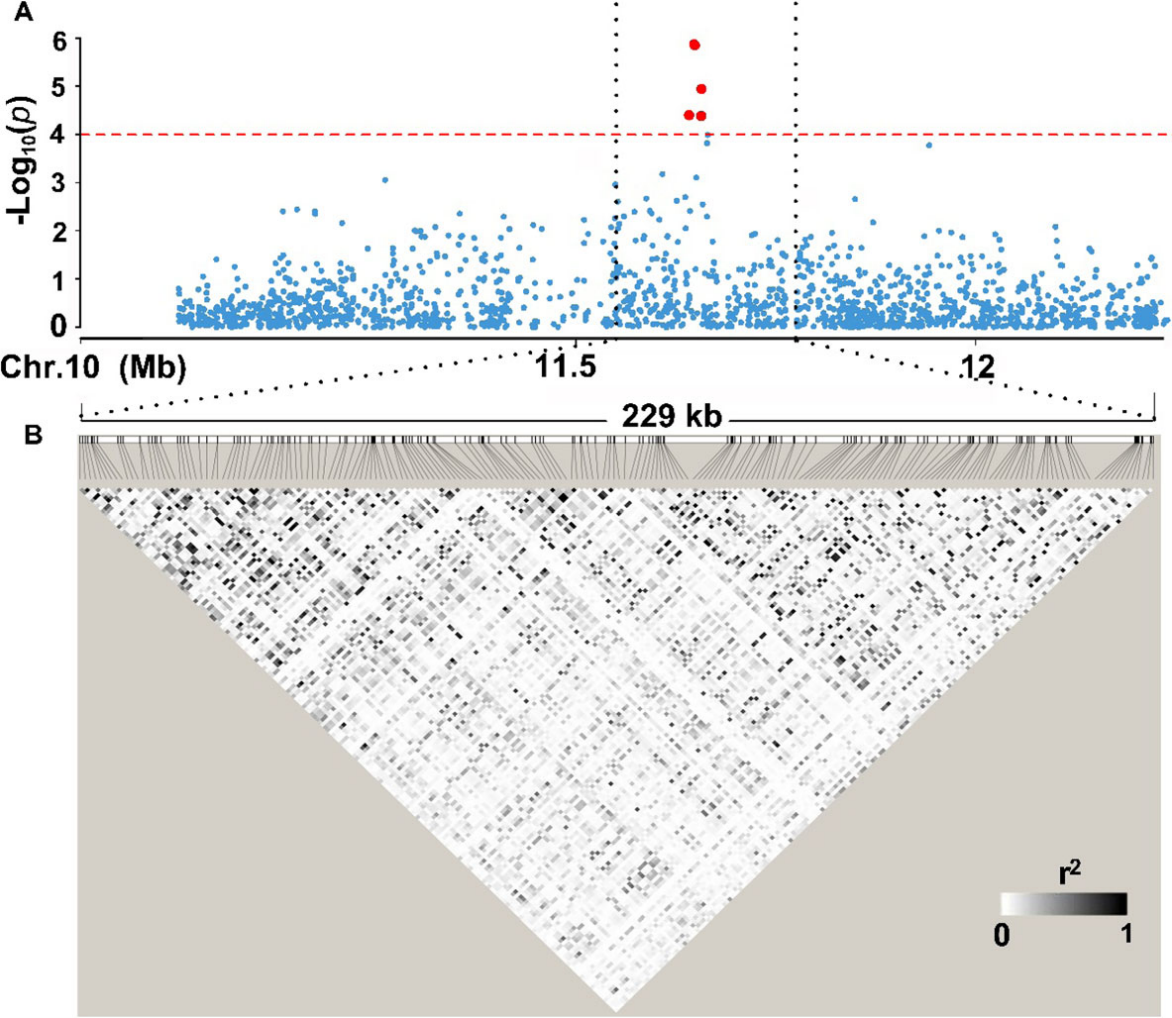
Candidate region estimation of *qLTG_sRDP2–10a* on chromosome 10. **a** Local Manhattan plot of GWAS for LTG. **b** LD heatmap around the peak

The LD decay analysis in the QTL region indicated that an approximately 229 kb region (from 11.551 Mb to 11.780 Mb on chromosome 10) at the associated locus was the putative region for *qLTG_sRDP2–10a* (Fig. 5). Based on release 7 of the MSU Rice Genome Annotation Project on rice IRGSP-1.0 genome (http://rice.plantbiology.msu.edu/) (Kawahara et al. 2013), there are 29 genes annotated within the region of *qLTG_sRDP2–10a*. To cut down the number of candidate genes, we selected four lines consisting of two high LTG lines (accession 517 from sRDP2 and S18) and two low LTG lines (accession 86 from sRDP2 and HJX74) based on haplotype analysis, to conducted DNA re-sequencing and RNA-seq. Firstly, we analyzed the coding sequence variation of the 29 genes in the 4 lines. Taking Nipponbare genome sequence as reference, a total of 144 variations (SNP, deletion or insertion) in coding regions were found, and only 47 variations in four genes, *LOC_Os10g22484*, *LOC_Os10g22510*, *LOC_Os10g22570* and *LOC_Os10g22600* exhibited consistent sequence differences between the high and low LTG lines. The SNP between high and low LTG lines in *LOC_Os10g22570* caused synonymous variation with no amino acid change, while the SNP in *LOC_Os10g22600* causes missense mutation resulting in amino acid substitution. For 41 sequence variations in *LOC_Os10g22484*, nine SNPs caused synonymous mutation and the other 32 sequence variations caused amino acid substitutions. The four sequence variations in *LOC_Os10g22510* also caused missense mutation and inframe deletion (Table S3, highlighted in yellow).

**Fig. 6:**
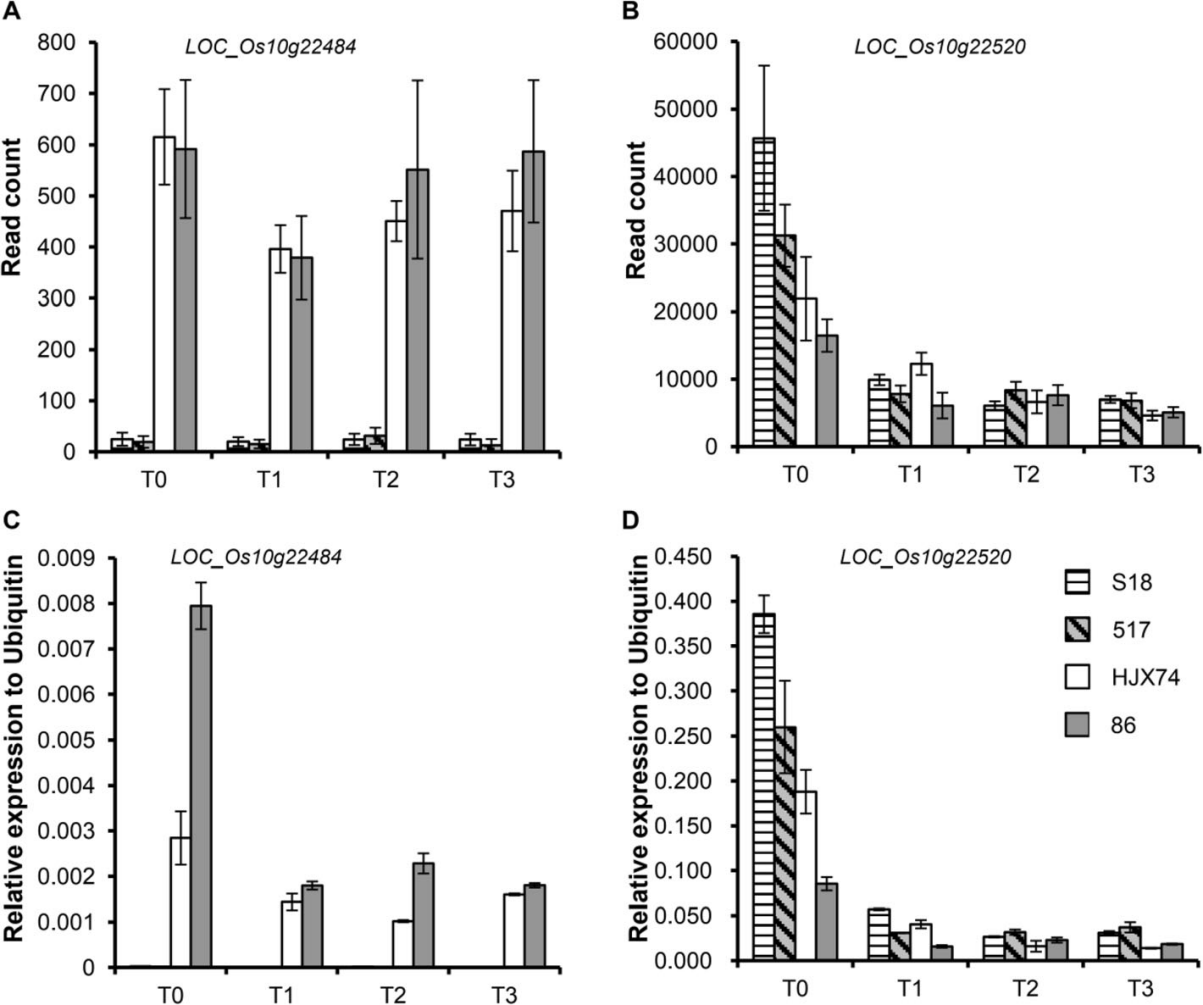
Temporal expression patterns of two candidate genes during germination in high LTG lines (S18 and 517) and low LTG lines (HJX74 and 86). **a** and **c** The expression patterns of *LOC_Os10g22484* measured by RNA-seq and qRT-PCR, respectively. **b** and **d** The expression patterns of *LOC_Os10g22520* measured by RNA-seq and qRT-PCR, respectively. T0 represents imbibition for 24 h; T1, T2 and T3 represent 13 °C cold treatment for 48 h, 96 h and 144 h, respectively

Simultaneously, gene differential expression analysis was conducted using these 4 lines, which were subjected to 13 °C low temperature treatment and sampled at 24 h after imbibition (T0) and 48 h (T1), 96 h (T2) and 144 h (T3) after cold treatment. RNA-seq revealed that 15 genes including *LOC_Os10g22600* were not expressed during germination (data not shown). For the other 14 genes, the expression of *LOC_Os10g22510* exhibited a similar pattern in high and low LTG lines. Only two genes, *LOC_Os10g22484* and *LOC_Os10g22520*, were differentially expressed between the two pairs of contrasting lines (Fig. 6a and b, Fig. S3). These results were further confirmed by qRT-PCR assays (Fig. 6c and d). Taken together, *LOC_Os10g22484* and *LOC_Os10g22520* were considered as the candidate gene for *qLTG_sRDP2–10a*. However, the expression patterns of the two candidate genes were different. The expression levels of *LOC_Os10g22484* in the high LTG lines were consistently significantly lower than that in the low LTG lines at all time points (*p* < 0.05), while *LOC_Os10g22520* only exhibited differential expression between the two pairs of contrasting lines at 24 h imbibition (T0), at which the high LTG lines exhibited significantly higher gene expression levels than the low LTG lines (*p* < 0.05).

**Fig. 7:**
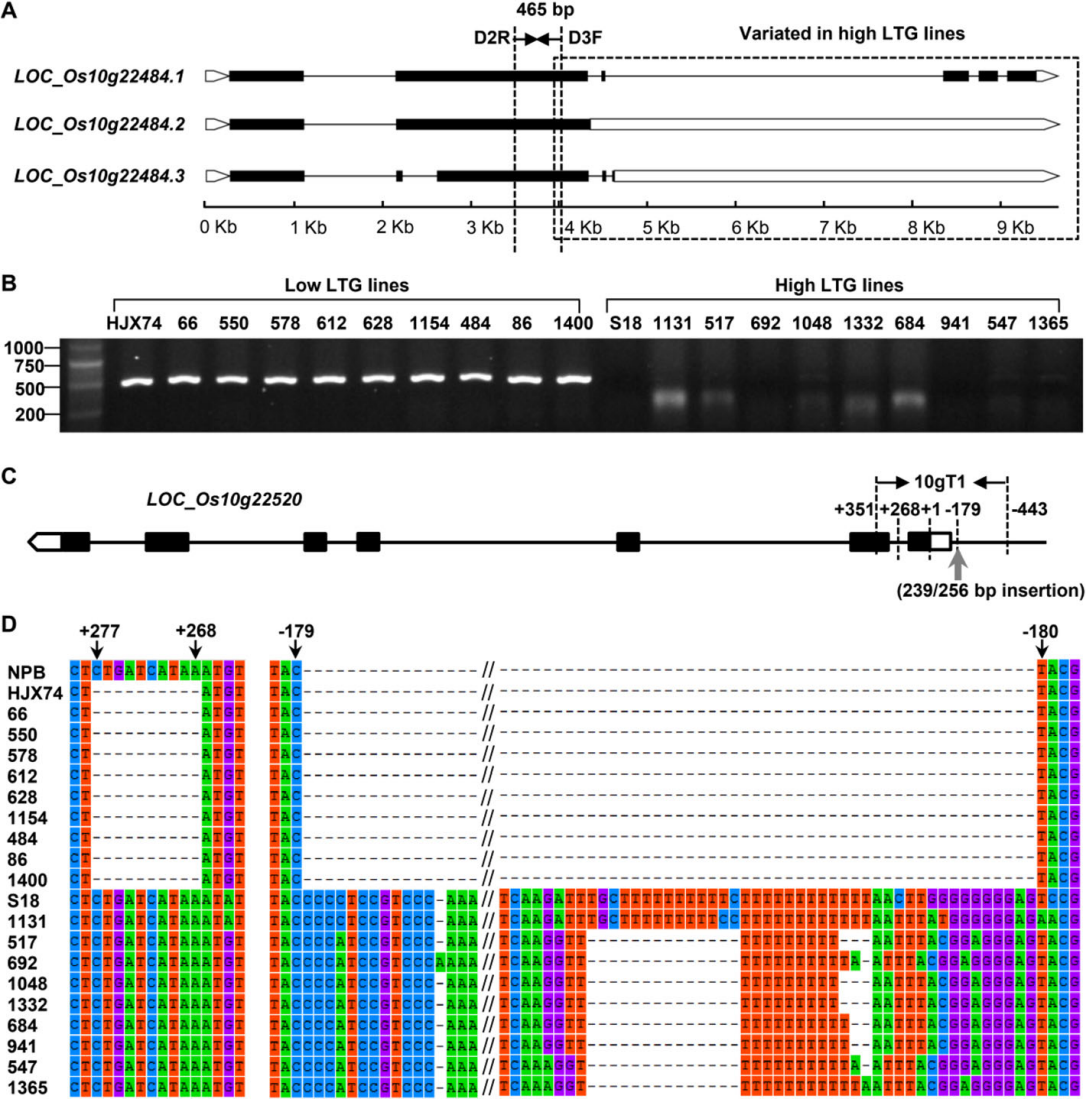
Sequence variations of *LOC_Os10g22484* and *LOC_Os10g22520* in high LTG and low LTG rice accessions. **a** Three predicted transcripts of *LOC_Os10g22484* from RGAP. Genomic analysis indicated that the sequences after 3969 bp were variated in 517 and S18, compared to 86 and HJX74. A pair of primers stretching across the variation joint were designed as D3F/D2R. **b** The amplified products of DNA from the selected 20 rice accessions using primer D3F/D2R revealed by agrose gel electrophoresis. **c** Gene structure of *LOC_Os10g22520*. + 1 indicates the site of initiation codon. Black rectangles, white rectangles and gray arrow indicate the exons, 3′ and 5′ UTR, and insertion sequence, respectively. A pair of primer was designed to amplify the sequence between + 351 to − 443 (designated as 10gT1). **d** Sequence alignment of 10gT1 in *LOC_Os10g22520* between high and low LTG rice accessions. The sequence of 10gT1 in Nipponbare (NPB) was taken as reference. The sequences from + 277 to + 268 were deleted in low LTG lines. A fragment of 239 or 256 bp was inserted after − 179 bp upstream of initiation codon in high LTG lines

To find the cause of expression variation of the two genes during imbibition and low temperature treatment, we compared the sequence differences of the two genes between the two pairs of contrasting lines based on DNA re-sequencing of all 4 lines above. The sequence comparisons revealed that there were insertion and deletion sequence differences in the promoter and the intron region of *LOC_Os10g22520* between the high and low LTG lines (Fig. 7c and Fig. S4), and an about 6 kb variation after 3969 bp at the 3′ end of *LOC_Os10g22484* in the high LTG lines (Fig. 7a and Fig. S4).

To confirm the relationship between the sequence variation of two genes and the LTG, we further compared the sequence differences of the two genes between 10 high LTG rice accessions and 10 low LTG rice accessions selected based on haplotype analysis, including the 4 lines above. The primers used to amplify and sequence the target regions of the genes were designed based on the released sequence of Nipponbare (Table S4). For the gene *LOC_Os10g22484*, primers (D3F/D2R) spanning the variation joint were designed to amplify the target regions of the selected 20 rice accessions (Fig. 7a). The agarose gel electrophoresis revealed that there were clear target bands in all low LTG rice accessions, but no amplified target band was observed in the high LTG rice accessions (Fig. 7b), suggesting that the sequence variations exist in high LTG accessions. For *LOC_Os10g22520*, primers were designed to amplify the sequence between + 351 to − 443 (designated as 10gT1) (Fig. 7c). The sequence comparisons revealed that there were insertion and deletion sequence differences in *LOC_Os10g22520* between the high and low LTG groups. A 239 bp or 256 bp insertion was observed at the position of − 179 in the promoter region of the high LTG rice accessions, while a 10 bp deletion was observed in the intron region (from the position of + 268 to + 277) in the low LTG rice accessions (Fig. 7d and Table S5).

## Discussion

### The *qLTG_sRDP2–10a* Has a Great Potential Value in Rice Breeding

In the present study, a total of eleven LTG QTLs were identified through GWAS. Among these QTLs, three QTLs, *qLTG_sRDP2–3/qLTG_JAP-3*, *qLTG_AUS-3* and *qLTG_sRDP2–12* are first reported in the present study, and the other 8 QTLs overlap with or are adjacent to the previously identified LTG QTLs from different studies (Table 1), suggesting reliability of the results and the diversity of the rice germplasm in this study. Notably, *qLTG_sRDP2–10a* identified in the present study overlaps with the LTG QTLs identified in the previous three studies (Jiang et al. 2006; Wang et al. 2018a, b). In the study conducted by Jiang et al. (2006), they used a F_2_ population derived from USSR5 (*japonica*, high LTG) and N22 (*indica*, low LTG), and 15 °C low temperature for mapping of LTG QTL. They mapped *qLTG-10* to 9.82–16.71 Mb on chromosome 10. Through GWAS, Wang et al. (2018a) identified a LTG QTL with the most significant SNP at the position of 11.57 Mb on chromosome 10 by using 187 rice accessions selected from the RDP1 under 12 °C low temperature. In another GWAS of LTG, Wang et al. (2018b) identified a LTG QTL with a significant SNP at the position of 11.89 Mb on chromosome 10 by using the Korean rice core set consisting of 137 accessions under 13 °C low temperature. Additionally, this QTL could be also detected using SSSL derived from GXN (high LTG) and HJX74 (low LTG) under 15 °C low temperature in the present study. Interestingly, *qCTSR10–1* for cold tolerance at the bud bursting stage with the most significant marker at 11.03 Mb (Zhang et al. 2018) and *qLTSS10–2* for cold tolerance at seedling stage with most significant marker at 11.62 Mb (Schläppi et al. 2017) were located in the same region as *qLTG_sRDP2–10a*. These QTLs located in the same region on the same chromosome might be attributed to the same QTL, pleiotropic effects of a gene, or the clustering of unrelated genes at this locus. Further studies are needed to elucidate these issues. But the same QTL for LTG was detected using different rice germplasm (USSR5, GXN, 375 rice accessions from RDP2, 187 rice accessions from RDP1, 137 accessions from Korean rice core set), different QTL detection approaches (bi-parental QTL analysis, GWAS and SSSL-based QTL analysis) and different low temperature conditions for cold treatment (12 °C for 10 days, 13 °C for 10 and 15 days, 15 °C for 10 and 13 days), suggesting that *qLTG_sRDP2–10a* is a stably expressed QTL for LTG. Moreover, *qLTG_sRDP2–10a* has the largest contribution to LTG variation in 375 rice accessions among the 9 LTG QTLs identified in the whole population. The SSSL-based QTL analysis indicated that the presence of *qLTG_sRDP2–10a* could result in 59.8% increase in LTG under 15 °C low temperature. Furthermore, our results suggest that *qLTG_sRDP2–10a* had no effect on the plant height, one of the main factors affecting lodging resistance in rice direct seeding practice. Large effect on LTG but no influence on plant height, and stable expression make *qLTG_sRDP2–10a* have a great potential value in rice breeding.

### *LOC_Os10g22484* and *LOC_Os10g22520* Could Be the Candidate Genes Underlying *qLTG_sRDP2–10a*

The germination process of rice seeds is complex and can be divided into three stages: imbibition, germination and bud bursting. With absorbing water at the imbibition stage, the metabolism inside the seed becomes active. The storage substances are converted to soluble substances such as monosaccharide and amino acids, and transported to the embryo cells, leading to rapid division and elongation of the embryonic cells. When its volume increases to a certain extent, the embryo breaks through the seed coat and continues to grow. Therefore, the genes responsible for germination should be involved in these processes. In the present study, four of 29 putative genes in *qLTG_sRDP2–10a* region exhibited consistent sequence variations in the coding regions between the high and low LTG lines, and the sequence variations of three genes, *LOC_Os10g22484*, *LOC_Os10g22510* and *LOC_Os10g22600* cause amino acid substitutions. Gene differential expression analysis revealed that *LOC_Os10g22600* were not expressed and the expression of *LOC_Os10g22510* exhibited a similar pattern in high and low LTG lines. Only the two genes, *LOC_Os10g22484* and *LOC_Os10g22520* exhibited differential expression at 24 h after imbibition, and only *LOC_Os10g22484* exhibited differential expression under 13 °C low temperature between the two pairs of lines with contrasting LTG (Fig. 6), suggesting that *LOC_Os10g22520* is responsible for seed imbibition, while *LOC_Os10g22484* is responsible for imbibition and germination at low temperatures. Furthermore, the sequence comparisons revealed a 239/256 bp insertion and 10 bp deletion in the promoter and the intron region of *LOC_Os10g22520* between the high and low LTG lines, and about 6 kb variation at the 3′ end of *LOC_Os10g22484* in the high LTG lines (Fig. 7). These results suggest that the sequence variations of *LOC_Os10g22484* and *LOC_Os10g22520* could be the cause for their differential expression in high and low LTG lines.

*LOC_Os10g22520* encodes a glycoside hydrolase. Glycoside hydrolases are a class of enzymes that hydrolyze glycosidic bonds in various carbohydrate-containing compounds by endo- or exo-cleavage to form monosaccharides, oligosaccharides or glycoconjugates. It is reported that glycoside hydrolases participate in the degradation of cell wall polysaccharides, and are involved in the control of plant cell wall loosening: regulation of germination, growth and development, fruit ripening, abscission, and cell adhesion (Minic and Jouanin 2006; Minic 2008). In the present study, *LOC_Os10g22520* exhibited significantly higher expression level in the high LTG lines than in low LTG lines, suggesting that it may be more active involvement in degradation of cell wall polysaccharides and cause tissue weakening, or involvement in carbohydrate mobilization to drive growth of the germinating embryo in high LTG lines, which consequently enhances their germinability at low temperature. *LOC_Os10g22484* encodes a NBS-LRR domain containing protein. So far, very limited information is available for the function of *LOC_Os10g22484*. The genome-wide gene expression profiling indicated that this gene was involved in development of panicle in rice (Ke et al. 2018) and also responded to submergence in rice (Xiong et al. 2012). Many NBS-LRR domain containing proteins function in plant defense response against pathogens (Caplan et al. 2008). It has been reported that there are crosstalks and balancing trade-offs between biotic stress and abiotic stress responses (Ku et al. 2018; Berens et al. 2019). For instance, plants exposed to abiotic stresses such as high salinity and drought often display reduced immune activity (Bostock et al. 2014). It could be possible that the sequence variation of the NBS-LRR domain containing protein gene, *LOC_Os10g22484* might result in increasing the germinability at low temperature in rice. To finalize the functions of these two genes on imbibitions and germination at low temperature in rice, transgenic experiments are underway.

### Integrating GWAS and SSSL Can Facilitate Identification of QTL for Complex Traits in Rice

With the rapid development of genome sequencing technology, GWAS has been widely applied for dissection of complex traits in rice and proved to be an efficient approach for QTL analysis (Huang et al. 2010; McCouch et al. 2016). However, there always exist false positive results in GWAS due to improper population structure and statistical methods, poor quality of genotyping, or phenotyping errors. Therefore, it is a routine to confirm the target QTLs before they can be used for breeding practice or advanced to functional gene cloning. Bi-parental population, such as a F_2_ or BC population is normally developed for confirmation of the results from GWAS. But this approach is low efficient because it takes more time and manpower for population development and QTL analysis. In the present study, we employed SSSL-based QTL analysis for confirmation of *qLTG_sRDP2–10a* from GWAS. Firstly, we screened the parents of SSSLs based on the haplotype of the target QTL (*qLTG_sRDP2–10a*) and their phenotypes of the target trait (LTG), and identified the right parents GXN and HJX74; then, screened SSSLs derived from GXN and HJX74 and identified the target SSSL S18 based on haplotype and the QTL region. Finally, QTL mapping was conducted simply by comparing the difference in LTG between the target SSSL S18 and its recurrent parent HJX74. Using this approach, we were able to confirm the existence of *qLTG_sRDP2–10a* and its phenotypic effect. Compared with the bi-parental QTL analysis, it saves a lot of time for population development, genotyping and phenotyping. Furthermore, the SSSL is a permanent line, it can be used for repeatedly experiments which is particularly important for complex traits like LTG. Therefore, the SSSL-based QTL analyses could be a simple and accurate approach for identification of QTL, and also a good complementary approach for confirmation of the results from GWAS. Therefore, integrating GWAS and SSSL can facilitate identification of QTL for complex traits in rice.

## Conclusion

In the present study, large LTG variations within 375 rice accessions from the RDP2 were observed, and the LTG of *Indica* group was significantly higher than that of *Japonica* and *Aus* groups (*p* < 0.01). Among the 11 LTG QTLs identified in this study, *qLTG_sRDP2–10a* had the largest contribution to LTG variations in 375 rice accessions and could be detected in five studies using different germplasm under different cold environments. Its large effect and stable expression make *qLTG_sRDP2–10a* particularly valuable in rice breeding. The two genes, *LOC_Os10g22484* and *LOC_Os10g22520* which exhibited differential expression and sequence variation between the high and low LTG lines were considered as the candidate genes underlying *qLTG_sRDP2–10a*. The sequence variations of the two genes could be the cause for their differential expression in high and low LTG lines. Our results suggest that integrating GWAS and SSSL can facilitate identification of QTL for complex traits in rice. The identification of *qLTG_sRDP2–10a* and its candidate genes provide a promising source for gene cloning of LTG and molecular breeding for LTG in rice.

## Methods

### Plant Materials

A subset of the RDP2 (sRDP2) consisting of 375 rice accessions from 56 countries were used for GWAS in this study (Table S1). These rice accessions were selected from the Rice Diversity Panel 2 (RDP2) consisting of 1568 rice accessions based on their diversity and origins (McCouch et al. 2016).

Single segment substitution line (SSSL) used for *qLTG_sRDP2–10a* validation was developed by backcross and marker-assisted selection (Zhang et al. 2004; Xi et al. 2006). Each SSSL contains only one chromosomal segment from Gangxiangnuo (GXN, the donor parent with high LTG), and has the background of Huajingxian74 (HJX74, the recurrent parent with low LTG).

All seeds used for LTG experiments in this study were stored at room temperature for 3 months after harvested.

### Evaluation of Low Temperature Germinabilty

Three replicates with 50 seeds per accession were adopted in evaluation of LTG in this study. The healthy and filled seeds were incubated at 49 °C for 96 h to break dormancy. After sterilized with 3% sodium hypochlorite solution, the seeds were soaked in distilled water for 24 h. Then, the soaked seeds were placed in a 7 cm petri dish with two layers of wet filter paper, and put into a growth chamber with a relative humidity of 80% for low temperature treatment for 10 days in dark. Low temperature of 13 °C and 15 °C were adopted in the sRDP2 and SSSLs, respectively. Emergence of plumule or radicle longer than 1 mm is considered as germination. The germination rates of the tested lines at low temperature (LT-GR) were estimated on the tenth day after cold treatment, and then the temperature in the growth chamber was increase to 30 °C for recovery. The germination rates of the tested lines at 30 °C (RT-GR) were estimated after 5 days. Low temperature germinability (LTG) of each line was expressed as the ratio of the germination rate at low temperature over the germination rate after recovery at 30 °C (LTG % = 100 x LT-GR / RT-GR).

### GWAS of sRDP2 and QTL Delimitation

GWAS analysis was performed as described in our previous study (Zhao et al. 2018) by using software GAPIT version 2 (Tang et al. 2016) and HDRA dataset consisting of 700 K single nucleotide polymorphisms (SNPs) (McCouch et al. 2016). SNPs were filtered by the criteria of having less than 30% missing data and minor allele frequency (MAF) > 0.05 (McCouch et al. 2016). In order to reduce the effect of population structure on GWAS, the mixed linear model (MLM) was selected in which the kinship matrix was used jointly with PC in GAPIT, and three PCs were included when analyzing across all subpopulations. Manhattan and QQ plots were produced using R package qqman (Turner 2018). A region having three or more than three significant SNPs (*p* < 0.0001) within 200 kb is considered as one QTL, which was used in previous studies (Shakiba et al. 2017;Wang et al. 2018a; Zhao et al. 2018; Jiang et al. 2019).

### SSSL-Based QTL Analysis

The detection of QTL in SSSLs was performed as described in our previous study (Yang et al. 2016). When the difference in LTG between the SSSL and its recurrent parent HJX74 was significant at *p*<0.05 based on *t*-test, a QTL for LTG was declared.

### RNA-Sequencing

Two high LTG accessions (517 from sRDP2 and S18) and two low LTG accessions (86 from sRDP2 and HJX74) were selected for RNA-sequencing based on haplotype analysis. Low temperature germination was conducted at 13 °C as described above. Sampling was done at 24 h after imbibition (T0), and 48 h (T1), 96 h (T2) and 144 h (T3) after cold treatment, respectively, with three biological replications. Total RNA was extracted from embryo (T0, T1 and T2) or embryo with coleoptile (T3) of germinating seed using Trizol reagent (Invitrogen, Carlsbad, CA, USA) and purified using RNeasy Plant Mini Kit (Qiagen, Valencia, CA). RNA-Seq was performed at Annoroad Gene Technology (Beijing, China), and data analysis was conducted using HISAT2-Stringtie-Deseq2 pipeline (Pertea et al. 2016). Raw counts of each sample exported from Stringtie were imported and normalized by DEseq2. Genes with read counts less than 10 in all samples were filtered out for further analysis. Then differentially expressed genes between two samples were identified according to the critiera of FDR ≤ 0.05 and estimated absolute log_2_ (FC) ≥ 1, which were exported from DEseq2 (Love et al. 2014).

### Differential Expression Analysis of Genes by qRT-PCR

RNA reverse transcription reactions were performed using the PrimeScript TM RT reagent kit (Takara, Japan). The primers for qRT-PCR were designed by Primer Premier 5.0. Real-time PCR was carried out using the SYBR Premix ExTaq TM kit (Takara, Japan), following the manufacturer’s instructions, on a Biorad CFX 96 Real-Time System. The ubiquitin was used as endogenous normalized genes for mRNA. The same RNA samples used in RNA-Seq assays were used to confirm the results of RNA-Seq, and all reactions were run in triplicate. Primers used to amplify the selected genes are listed in Table S4.

### DNA Re-Sequencing

The leaves of rice seedlings were collected and subjected to DNA extraction by the CTAB method. The experimental procedure was performed in accordance with standard protocols provided by Illumina company. The qualified genomic DNA was fragmented by ultrasonication, and then the fragmented DNA was subjected to fragment purification, terminal repair, 3′ end plus A, ligation and sequencing, and then fragmentation by agarose gel electrophoresis. The DNA size was selected and PCR amplification was performed to form a sequencing library. The constructed library was first subjected to library quality inspection, and the quality-qualified library was sequenced using Hiseq 4000(Illumina company, USA).

### Data Analysis

A phylogenetic tree of 375 rice accessions was constructed by MEGA 7.0 (Kumar et al. 2016) using SNP data mentioned above. The population structure of 375 rice accessions was constructed by Admixture v1.3.0 (http://software.genetics.ucla.edu/admixture/) using the appropriate format generated from PLink v1.9 (Shaun et al. 2007). Preset ancestry number (K, from one to nine) was applied to ancestry tracing analysis of these accessions. Pairwise relative kinship and PCA were generated in GAPIT version 2 (Tang et al. 2016). LD heatmap was drawn with Haploview (Barrett et al. 2005). A *t*-test was conducted using SAS (SAS Institute 2000) to detect the differences in LTG and expression levels of the candidate genes between the selected rice accessions.

## Supplementary information

**Additional file 1: Figure S1.** Population structure analysis of sRDP2 consisting of 375 rice accessions. A, Clustering of the 375 rice accessions according to their genotypes determined by 700 K SNPs. B, Genetic structure of sRDP2. Subgroups (*K* = 3) inferred using Admixture software. C, Pair-wise plots of principal component analysis of sRDP2. D, The heat map of pair-wise relative kinship analysis of sRDP2.

**Additional file 2: Figure S2.** The plant height of HJX74 and S18 in the field. The observation value was the average of three replicates, with 20 plants in each replicate.

**Additional file 3: Figure S3.** Temporal expression patterns of the other 12 putative genes within the region of *qLTG-sRDP2–10a* measured by RNA-seq. T0 represents imbibition for 24 h; T1, T2 and T3 represent 13 °C cold treatment for 48 h, 96 h and 144 h, respectively.

**Additional file 4: Figure S4.** Gene structure variation analysis of *LOC_Os10g22484* and *LOC_Os10g22520* based on the DNA re-sequencing.

**Additional file 5: Table S1.** The LTG and information of 375 rice accessions.

**Additional file 6: Table S2.** The rice accessions with LTG over 90%.

**Additional file 7: Table S3.** The coding sequence variation analysis of genes located in *qLTG_sRDP2–10a* region between high LTG lines (517 and S18) and low LTG lines (HJX74 and 86) based on DNA re-sequencing.

**Additional file 8: Table S4.** Primer sequences used in this study.

**Additional file 9: Table S5.** Sequence differences of 10gT1 in *LOC_Os10g22520* between high and low LTG rice accessions.

## Data Availability

The datasets supporting the conclusions of this article are provided within the article and its additional files.

## Abbreviations

LTG: Low temperature germinability
QTLs: Quantitative trait loci
SSSL: Single segment substitution line
GWAS: Genome-wide association study
LD: Linkage disequilibrium
MAF: Minor allele frequency
NBS-LRR: Nucleotide binding site-leucine rich repeat
SNP: Single-nucleotide polymorphism
RDP2: Rice Diversity Panel 2
HJX74: Huajingxian74
GXN: Ganxiangnuo
