# Supplementary material for: The Candidate Genes Underlying a Stably Expressed QTL for Low Temperature Germinability in Rice (Oryza sativa L.)
Source: Rice (N Y). 2020 Oct 19;13:74. doi: 10.1186/s12284-020-00434-z (PMC7573065; doi:10.1186/s12284-020-00434-z)
Supplement: Supplementary file 1 — Additional file 1: Figure S1. Population structure analysis of sRDP2 consisting of 375 rice accessions. A, Clustering of the 375 rice accessions according to their genotypes determined by 700 K SNPs. B, Genetic structure of sRDP2. Subgroups (K = 3) inferred using Admixture software. C, Pair-wise plots of principal component analysis of sRDP2. D, The heat map of pair-wise relative kinship analysis of sRDP2. [file 12284_2020_434_MOESM1_ESM.docx]

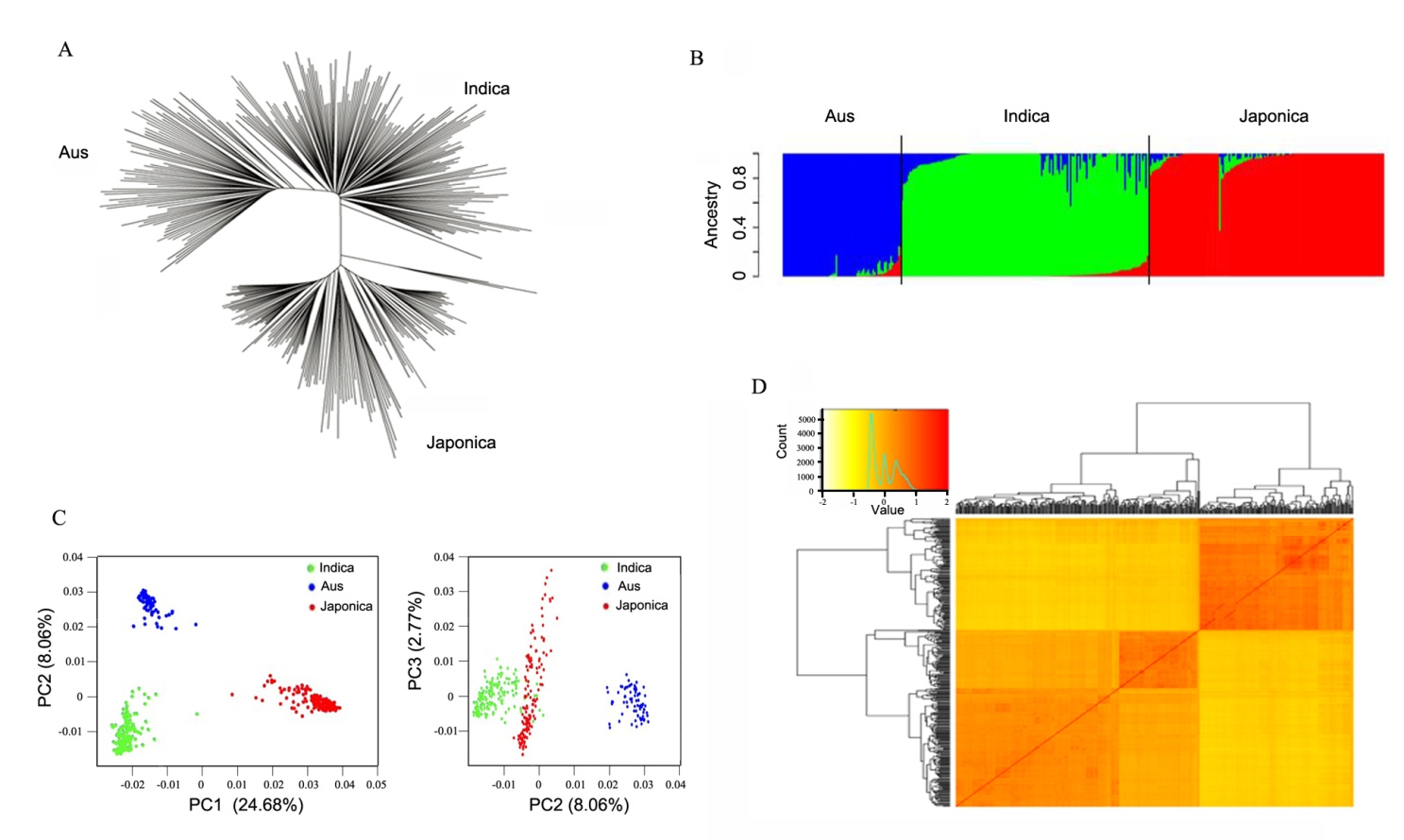


Figure S1 Population structure analysis of sRDP2 consisting of 375 rice accessions.

A, Clustering of the 375 rice accessions according to their genotypes determined by 700K SNPs. B, Genetic structure of sRDP2. Subgroups (K = 3) inferred using Admixture software. C, Pair-wise plots of principal component analysis of sRDP2. D, The heat map of pair-wise relative kinship analysis of sRDP2.
