# Supplementary material for: The Candidate Genes Underlying a Stably Expressed QTL for Low Temperature Germinability in Rice (Oryza sativa L.)
Source: Rice (N Y). 2020 Oct 19;13:74. doi: 10.1186/s12284-020-00434-z (PMC7573065; doi:10.1186/s12284-020-00434-z)
Supplement: Supplementary file 2 — Additional file 2: Figure S2. The plant height of HJX74 and S18 in the field. The observation value was the average of three replicates, with 20 plants in each replicate. [file 12284_2020_434_MOESM2_ESM.docx]

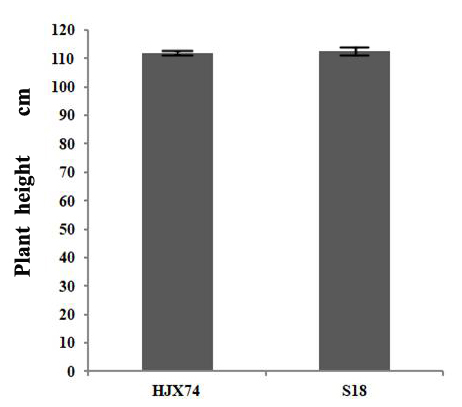


Figure S2 The plant height of HJX74 and S18 in the field.

The observation value was the average of three replicates, with 20 plants in each replicate.
