# Supplementary material for: The Candidate Genes Underlying a Stably Expressed QTL for Low Temperature Germinability in Rice (Oryza sativa L.)
Source: Rice (N Y). 2020 Oct 19;13:74. doi: 10.1186/s12284-020-00434-z (PMC7573065; doi:10.1186/s12284-020-00434-z)
Supplement: Supplementary file 3 — Additional file 3: Figure S3. Temporal expression patterns of the other 12 putative genes within the region of qLTG-sRDP2–10a measured by RNA-seq. T0 represents imbibition for 24 h; T1, T2 and T3 represent 13 °C cold treatment for 48 h, 96 h and 144 h, respectively. [file 12284_2020_434_MOESM3_ESM.docx]

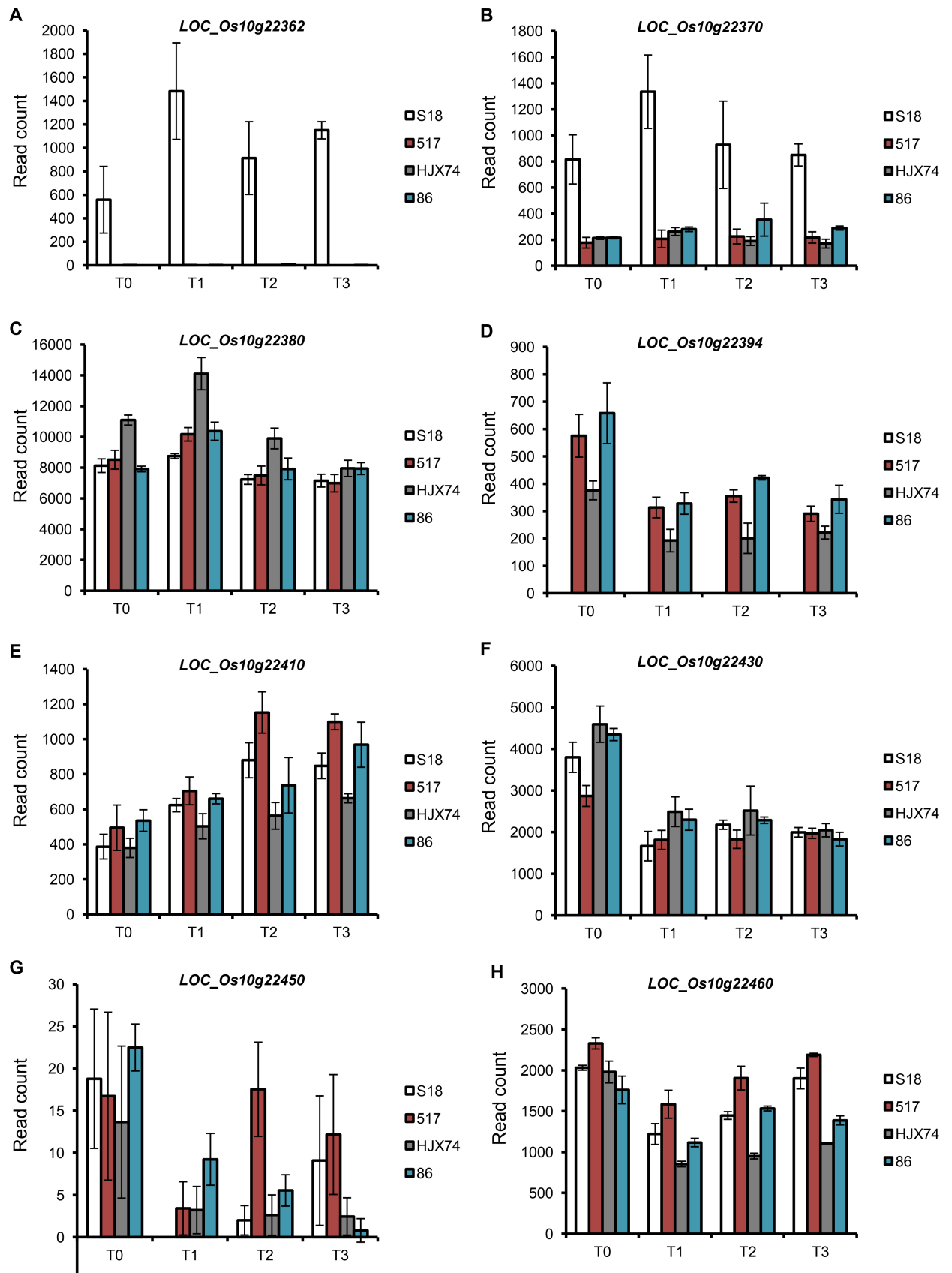

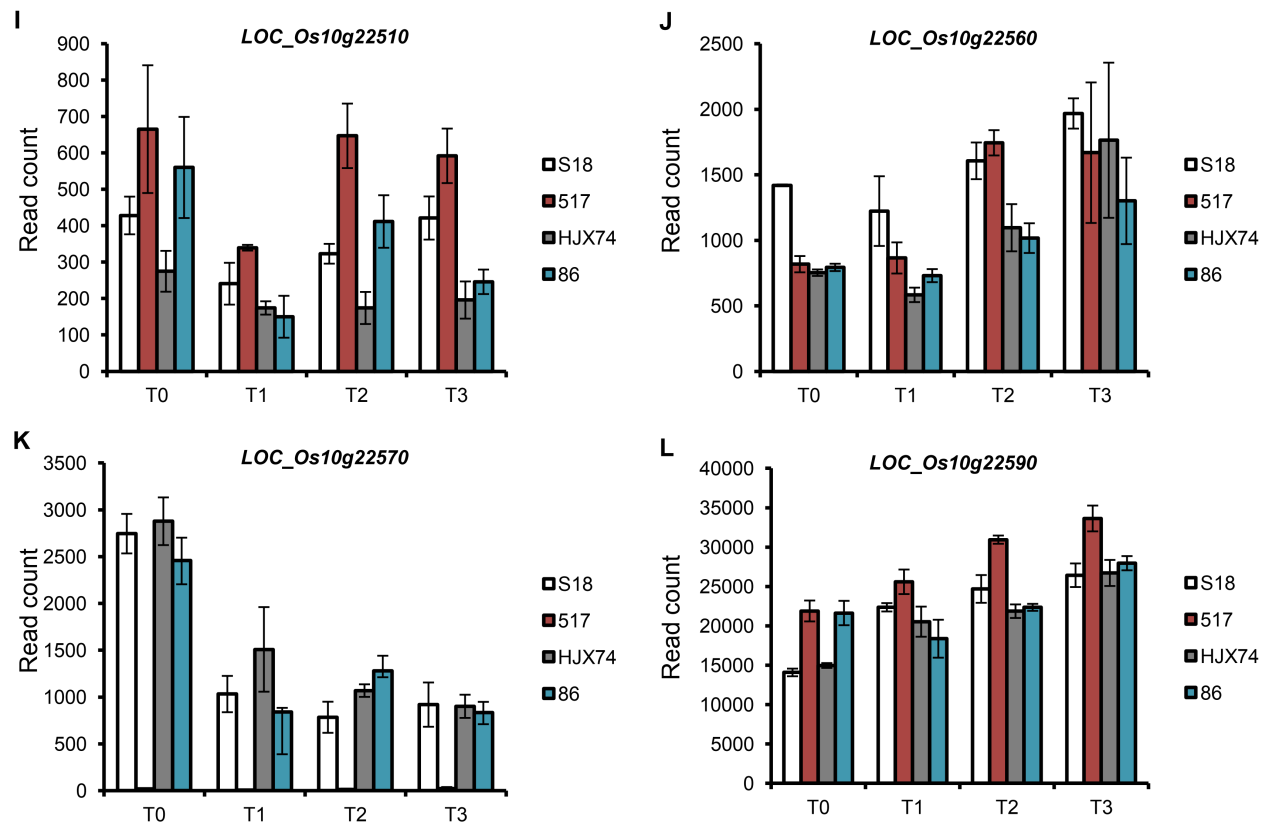


Figure S3 Temporal expression patterns of the other 12 putative genes within the region of qLTG-sRDP2-10a measured by RNA-seq. T0 represents imbibition for 24 h; T1, T2 and T3 represent 13℃ cold treatment for 48 h, 96 h and 144 h, respectively.
