# Supplementary figures and images for: The Candidate Genes Underlying a Stably Expressed QTL for Low Temperature Germinability in Rice (Oryza sativa L.)
Source: Rice (N Y). 2020 Oct 19;13:74. doi: 10.1186/s12284-020-00434-z (PMC7573065; doi:10.1186/s12284-020-00434-z)

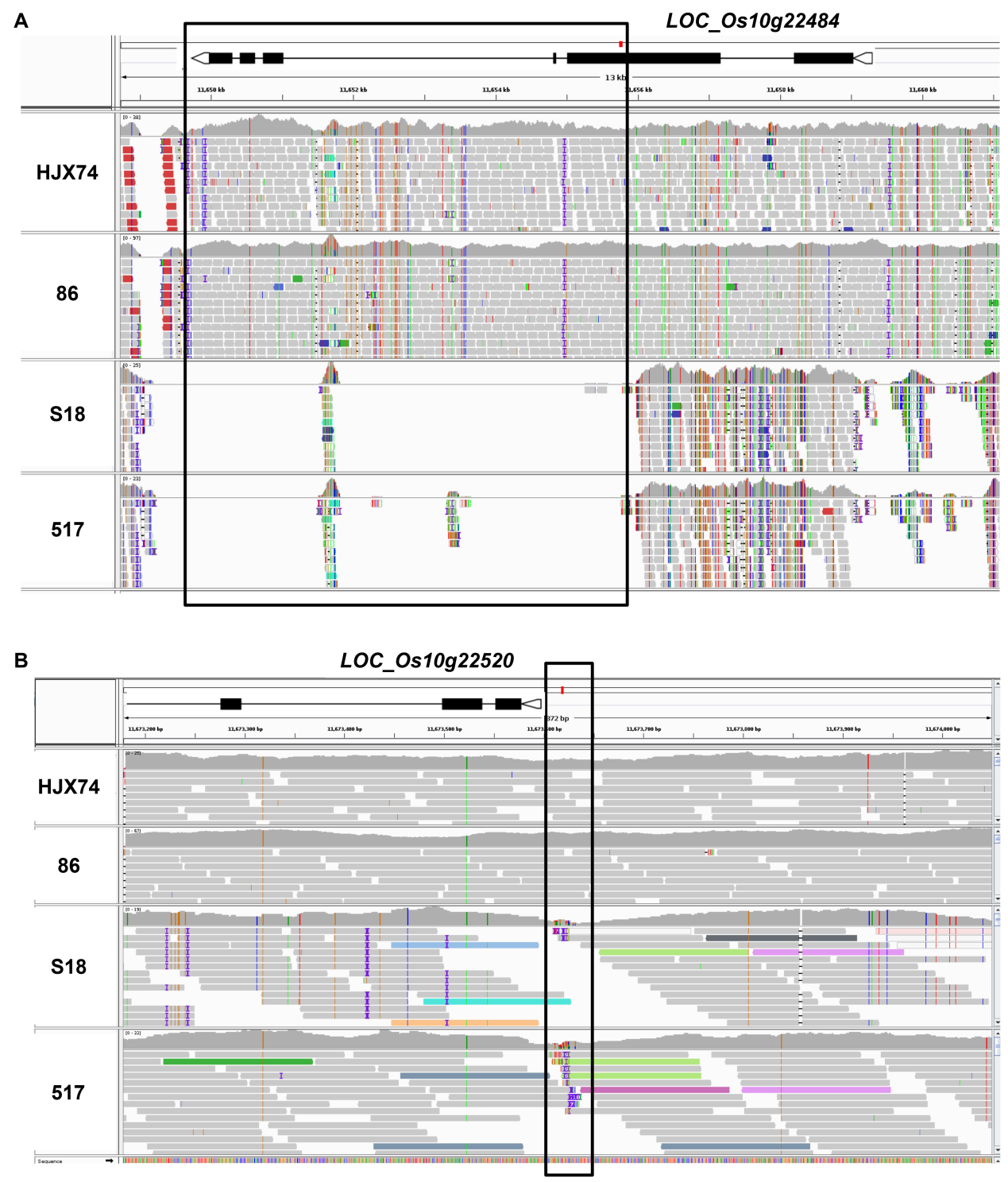


Figure S4 Gene structure variation analysis of *LOC_Os10g22484* and *LOC_Os10g22520* based on the DNA re-sequencing.

Supplement: Supplementary file 4 — Additional file 4: Figure S4. Gene structure variation analysis of LOC_Os10g22484 and LOC_Os10g22520 based on the DNA re-sequencing. [file 12284_2020_434_MOESM4_ESM.docx]
