# Supplementary material for: The Candidate Genes Underlying a Stably Expressed QTL for Low Temperature Germinability in Rice (Oryza sativa L.)
Source: Rice (N Y). 2020 Oct 19;13:74. doi: 10.1186/s12284-020-00434-z (PMC7573065; doi:10.1186/s12284-020-00434-z)
Supplement: Supplementary file 9 — Additional file 9: Table S5. Sequence differences of 10gT1 in LOC_Os10g22520 between high and low LTG rice accessions. [file 12284_2020_434_MOESM9_ESM.docx]

Table S5. Sequence differences of 10gT1 in *LOC_Os10g22520* between high and low LTG rice accessions

| Variety/Accession number | Sequence |
| --- | --- |
| Nipponbare | CAGGTACATGTTCTGCGTCACCGACTTGAATTGCAGCTGGGTTCCATCCTAAAATGCATAGAAAAGCGTGTTCTCTGATCATAAATGTTTGATGTTTAGAATAAGATTTGATAAGATTTTTGAAGTTTTTAATCATCCAAATGTACTTACCAGGAGGTCTTTGTTTGGAATGTCGTCGAAGAGGGAGGGAAGGATCCAGCCCTCCGTGACGAGCCAACCTCCAAGGTTCACCGCCCGAATGGGGGGCGACGGGGCGCCCCGTTTCGCGGGCCGCACCGTCCTCCCATCGGAGACTGAGAAGAGGCACGGCGAGCAGAGGAGGAGGAAGAGGAGGAGCTCAAAATGGCGCATGATGAAGACGAAGATTTTTCAGCTCTTGATGACTATCTTAGCTCAGCTTGCCAATGTATATATATACACAACGTAGTGCAGCGATCGATGCATGTGCGTGTACGAACTGCGTAATTAATGGATAGACGTGCGTGCTTAGAATAAAAATGTGGACATCCAGAGTTGAATAGCTTGATGCCTAC*GTGCGACGTTGTTGGCGTCCTGCACGCTTAATAATGAAGCTGCTTTGACGTTTGACCGCAGCCGTCCGATCGATGGTGCGGCTGGCCGGATGCCATTTGGTTTGACCCGTCAAACTCCGCCGGTGGCCGTCGGATGCTCATCGAACGGATATCGCATCGATCTTCAGATAATCGTCAGATTGGATAACGTATACGGATTAGTTCCGATCCAGGGGTGTGGATTCAGCGGCGCGGGCAAATTCTATTGGCTGCGTGAGCTTC |
| S18 | TCATCCTAAATGCATAGAACAGCGTGTTCTCTGATCATAAATATTTGATGTTTAGAATAAGATTTGATAAGATTTTTGAAGCTTTTTGATCGTCGGAATGTGCTTATACCAGGAGGTCTTTGTTTGGAATGTCGTCGAAGAGGGAGGGAAGGATCCAGCCCTCCGTGACGAGCCACCCTCCGAGGTTCACCGCCCGAATGGGGGGCAACGGGGCGCCCTGTTTCGCGGGCCGCACCGTCCTCCCATCGGAGACGGAGAAGAGGCACGGCGAGCAGAGGAGGAGGAAGAGGAGGAGGAGCTCGAAATGGCGCATGATGAAGACGAAGATTCTTCAGCTCTTGATGACTATCTTAGCTCAGCTTGCCAATGTATATATATATACACAACGTAATGCAGCGATCGATGCATGTGAGTGTACGAACTGCGTAATTAATGGATAGACGTGCGTGCTTAGAATAAAAATGTGGACATCCAGAGTTGAATAGCTTGATGCCTACCCCCTCCGTCCCAAAAAAAAAGATAAACCCTAGTTTCCGTGCCCAACGTTTGACCGTCCGTCTTATTTGAAAAAATTATAAAAAAAATTAAAAAGATAAGTCACGCATAAAATATTAATCATGTTTTATCATCTAACAATAATAAAAATACGAATTATAAAAAAATTTCATATAATACGGACAGTTAAAGTTGGATACCGAAACTCAAGATTTGCTTTTTTTTTTCTTTTTTTTTTTTAACTTGGGGGGGGAGTCCGGGCAACTTTTTTGGGCGCCCGGCCCGCTAAAAAAGGAACCGGCTTTGACGTTGACCGCACCCGCCCAATCAAGGGGGCGGCTGGCC |
| 1131 | ATCCTAGATGCATAGAACAGCGTGTTCTCTGATCATAAATATTTGATGTTTAGAATAAGATTTGATAAGATTTTTGAAGCTTTTTGATCGTCGGAATGTGCTTATACCAGGAGGTCTTTGTTTGGAATGTCGTCGAAGAGGGAGGGAAGGATCCAGCCCTCCGTGACGAGCCACCCTCCGAGGTTCACCGCCCGAATGGGGGGCAACGGGGCGCCCTGTTTCGCGGGCCGCACCGTCCTCCCATCGGAGACGGAGAAGAGGCACGGCGAGCAGAGGAGGAGGAAGAGGAGGAGGAGCTCGAAATGGCGCATGATGAAGACGAAGATTCTTCAGCTCTTGATGACTATCTTAGCTCAGCTTGCCAATGTATATATATATACACAACGTAATGCAGCGATCGATGCATGTGAGTGTACGAACTGCGTAATTAATGGATAGACGTGCGTGCTTAGAATAAAAATGTGGACATCCAGAGTTGAATAGCTTGATGCCTACCCCCTCCGTCCCAAAAAAAAAGATAAACCCTAGTTTCCGTGCCCAACGTTTGACCGTCCGTCTTATTTGAAAAAATTATAAAAAAAATTAAAAAGATAAGTCACGCATAAAATATTAATCATGTTTTATCATCTAACAATAATAAAAATACGAATTATAAAAAAATTTCATATAATACGGACAGTTAAAGTTGGATACCGAAACTCAAGATTTGCTTTTTTTTTCCTTTTTTTTTTTTAATTTATGGGGGGAGAACGGGCAACGTTTTGGGGCCCGGCCGCTAAAAAAGGAACCGGCTTTGACTTTTGACCGCCCCGCCCAACCGAGGGGGGGGGGGGCGGGGGCCTTTGGTTT |
| 517 | TGCATAGAAAGCGTGTTCTCTGATCATAAATGTTTGATGTTTAGAATAAGATTTGATAAGATTTTTGAAGTTTTTAATCATCCAAATGTACTTACCAGGAGGTCTTTGTTTGGAATGTCGTCGAAGAGGGAGGGAAGGATCCAGCCCTCCGTGACGAGCCAACCTCCGAGGTTCACCGCCCGAATGGGGGGCGACGGGGCGCCCCGTTTCGCGGGCCGCACCGTCCTCCCATCGGAGACTGAGAAGAGGCACGGCGAGCAGAGGAGGAGGAAGAGGAGGAGCTCAAAATGGCGCATGATGAAGACGAAGATTTTTCAGCTCTTGATGACTATCTTAGCTCAGCTTGCCAATGTATATATATACACAACGTAATGCAGCGATCGATGCATGTGCGTGTACGAACTGCGTAATTAATGGATAGACGTGCGTGCTTAGAATAAAAATGTGGACATCCAGAGTTGAATAGCTTGATGCCTACCCCATCCGTCCCAAAAAAAAAGACAAACCCTAATTTCCGTGCTCAACGTTTGACCGTCCGTCTTATTTGAAAAAATTATGAAAAAAATTAAAAAGACAAGTCACGCATAAAATATTAATCATGTTTTATCATCTAACAATAATAAAAATACGAATTATAAAAAAATTTCATATAATACGTACAGTTAAAGTTGGACACCAAAACTCAAGGTTTTTTTTTTTAATTTACGGAGGGAGTACGTGCGACGTTGTTGGCGTCCTGCACGCTTAATAATGAAGCTGCTTTGACGTTTGACCGCAGCCGTCCGATCGATGGTGCGGCTGGCCGGATGCCATTTGGTTTGACCCGTCAAACTCCGCCGGTGGCCGTCGGATGCTCATCGAACGGATATCGCATCGATC |
| 692 | TCCTAAATGCATAGAAAGCGTGTTCTCTGATCATAAATGTTTGATGTTTAGAATAAGATTTGATAAGATTTTTGAAGTTTTTAATCATCCAAATGTACTTACCAGGAGGTCTTTGTTTGGAATGTCGTCGAAGAGGGAGGGAAGGATCCAGCCCTCCGTGACGAGCCAACCTCCGAGGTTCACCGCCCGAATGGGGGGCGACGGGGCGCCCCGTTTCGCGGGCCGCACCGTCCTCCCATCGGAGACTGAGAAGAGGCACGGCGAGCAGAGGAGGAGGAAGAGGAGGAGCTCAAAATGGCGCATGATGAAGACGAAGATTTTTCAGCTCTTGATGACTATCTTAGCTCAGCTTGCCAATGTATATATATACACAACGTAATGCAGCGATCGATGCATGTGCGTGTACGAACTGCGTAATTAATGGATAGACGTGCGTGCTTAGAATAAAAATGTGGACATCCAGAGTTGAATAGCTTGATGCCTACCCCATCCGTCCCAAAAAAAAAAGACAAACCCTAGTTTCCGTGCTCAACGTTTGACCGTCCGTCTTATTTGAAAAAATTATGAAAAAAATTAAAAAGACAAGTCACGCATAAAATATTAATCATGTTTTATCATCTAACAATAATAAAAATACGAATTATAAAAAAATTTCATATAATACGTACGGTTAAAGTTGGACACCAAAACTCAAGGTTTTTTTTTTTTAATTTACGGAGGGGAGTACGTGCGACGTTGTTGGCGTCCTGCACGCTTAATAATGAAGCTGGCTTTGACGTTTGACCGCAGCGTCGATCGATGGTGCCGCTGCGATGCCATTGTTTGACCGTCAACTCCGCCGGGTGCCGTTGGATC |
| 1048 | CATCCTAAATGCATAGAAAGCGTGTTCTCTGATCATAAATGTTTGATGTTTAGAATAAGATTTGATAAGATTTTTGAAGTTTTTAATCATCCAAATGTACTTACCAGGAGGTCTTTGTTTGGAATGTCGTCGAAGAGGGAGGGAAGGATCCAGCCCTCCGTGACGAGCCAACCTCCGAGGTTCACCGCCCGAATGGGGGGCGACGGGGCGCCCCGTTTCGCGGGCCGCACCGTCCTCCCATCGGAGACTGAGAAGAGGCACGGCGAGCAGAGGAGGAGGAAGAGGAGGAGCTCAAAATGGCGCATGATGAAGACGAAGATTTTTCAGCTCTTGATGACTATCTTAGCTCAGCTTGCCAATGTATATATATACACAACGTAATGCAGCGATCGATGCATGTGCGTGTACGAACTGCGTAATTAATGGATAGACGTGCGTGCTTAGAATAAAAATGTGGACATCCAGAGTTGAATAGCTTGATGCCTACCCCATCCGTCCCAAAAAAAAAGACAAACCCTAATTTCCGTGCTCAACGTTTGACCGTCCGTCTTATTTGAAAAAATTATGAAAAAAATTAAAAAGACAAGTCACGCATAAAATATTAATCATGTTTTATCATCTAACAATAATAAAAATACGAATTATAAAAAAATTTCATATAATACGTACAGTTAAAGTTGGACACCAAAACTCAAGGTTTTTTTTTTTAATTTACGGAGGGAGTACGTGCGACGTTGTTGGCGTCCTGCACGCTTAATAATGAAGCTGCTTTGACGTTTGACCGCAGCCGTCCGATCGATGGTGCGGCTGGCCGGATGCCATTTGGTTTGACCCGTCAACTCCGCCGGTGGCCGTCGGATGCTCATCGAACGGATATCGCATCGATCTCAG |
| 1332 | ATGCATAGAAAGCGTGTTCTCTGATCATAAATGTTTGATGTTTAGAATAAGATTTGATAAGATTTTTGAAGTTTTTAATCATCCAAATGTACTTACCAGGAGGTCTTTGTTTGGAATGTCGTCGAAGAGGGAGGGAAGGATCCAGCCCTCCGTGACGAGCCAACCTCCGAGGTTCACCGCCCGAATGGGGGGCGACGGGGCGCCCCGTTTCGCGGGCCGCACCGTCCTCCCATCGGAGACTGAGAAGAGGCACGGCGAGCAGAGGAGGAGGAAGAGGAGGAGCTCAAAATGGCGCATGATGAAGACGAAGATTTTTCAGCTCTTGATGACTATCTTAGCTCAGCTTGCCAATGTATATATATACACAACGTAATGCAGCGATCGATGCATGTGCGTGTACGAACTGCGTAATTAATGGATAGACGTGCGTGCTTAGAATAAAAATGTGGACATCCAGAGTTGAATAGCTTGATGCCTACCCCATCCGTCCCAAAAAAAAAGACAAACCCTAATTTCCGTGCTCAACGTTTGACCGTCCGTCTTATTTGAAAAAATTATGAAAAAAATTAAAAAGACAAGTCACGCATAAAATATTAATCATGTTTTATCATCTAACAATAATAAAAATACGAATTATAAAAAAATTTCATATAATACGTACAGTTAAAGTTGGACACCAAAACTCAAGGTTTTTTTTTTTAATTTACGGAGGGAGTACGTGCGACGTTGTTGGCGTCCTGCACGCTTAATAATGAAGCTGCTTTGACGTTTGACCGCAGCCGTCCGATCGATGGTGCGGCTGGCCGGATGCCATTTGGTTTGACCCGTCAAACTCCGCCGGTGGCCGTCGGATGCTCTCGAACGGATACGCATCGATCT |
| 684 | TCCTAAAATGCATAGAAAGCGTGTTCTCTGATCATAAATGTTTGATGTTTAGAATAAGATTTGATAAGATTTTTGAAGTTTTTAATCATCCAAATGTACTTACCAGGAGGTCTTTGTTTGGAATGTCGTCGAAGAGGGAGGGAAGGATCCAGCCCTCCGTGACGAGCCAACCTCCGAGGTTCACCGCCCGAATGGGGGGCGACGGGGCGCCCCGTTTCGCGGGCCGCACCGTCCTCCCATCGGAGACTGAGAAGAGGCACGGCGAGCAGAGGAGGAGGAAGAGGAGGAGCTCAAAATGGCGCATGATGAAGACGAAGATTTTTCAGCTCTTGATGACTATCTTAGCTCAGCTTGCCAATGTATATATATACACAACGTAATGCAGCGATCGATGCATGTGCGTGTACGAACTGCGTAATTAATGGATAGACGTGCGTGCTTAGAATAAAAATGTGGACATCCAGAGTTGAATAGCTTGATGCCTACCCCATCCGTCCCAAAAAAAAAGACAAACCCTAATTTCCGTGCTCAACGTTTGACCGTCCGTCTTATTTGAAAAAATTATGAAAAAAATTAAAAAGACAAGTCACGCATAAAATATTAATCATGTTTTATCATCTAACAATAATAAAAATACGAATTATAAAAAAATTTCATATAATACGTACAGTTAAAGTTGGACACCAAAACTCAAGGTTTTTTTTTTTTAATTTACGGAGGGAGTACGTGCGACGTTTGTTGGCGTCCTGCACGCTTTAATAATGAAGCTGCTTTGACGGTTTGACCGCAGCGTCGATCGATGGTGCGGCTTACCGGAATGGCATTGTTGAACCGTCCAACTCCGCCGGTACCGTCA |
| 941 | TCCTAAAATGCATAGAAAGCGTGTTCTCTGATCATAAATGTTTGATGTTTAGAATAAGATTTGATAAGATTTTTGAAGTTTTTAATCATCCAAATGTACTTACCAGGAGGTCTTTGTTTGGAATGTCGTCGAAGAGGGAGGGAAGGATCCAGCCCTCCGTGACGAGCCAACCTCCGAGGTTCACCGCCCGAATGGGGGGCGACGGGGCGCCCCGTTTCGCGGGCCGCACCGTCCTCCCATCGGAGACTGAGAAGAGGCACGGCGAGCAGAGGAGGAGGAAGAGGAGGAGCTCAAAATGGCGCATGATGAAGACGAAGATTTTTCAGCTCTTGATGACTATCTTAGCTCAGCTTGCCAATGTATATATATACACAACGTAATGCAGCGATCGATGCATGTGCGTGTACGAACTGCGTAATTAATGGATAGACGTGCGTGCTTAGAATAAAAATGTGGACATCCAGAGTTGAATAGCTTGATGCCTACCCCATCCGTCCCAAAAAAAAAGACAAACCCTAATTTCCGTGCTCAACGTTTGACCGTCCGTCTTATTTGAAAAAATTATGAAAAAAATTAAAAAGACAAGTCACGCATAAAATATTAATCATGTTTTATCATCTAACAATAATAAAAATACGAATTATAAAAAAATTTCATATAATACGTACAGTTAAAGTTGGACACCAAAACTCAAGGTTTTTTTTTTTTAATTTACGGAGGGAGTACGTGCGACGTTTGTTGGCGTCCTGCACGCTTTAATAATGAAGCTGCTTTGACGGTTTGACCGCAGCGTCGATCGATGGTGCGGCTTACCGGAATGGCATTGTTGAACCGTCCAACTCCGCCGGTACCGTCA |
| 547 | AAGGAGGCTGGACTTCATCCTATATGCATAGAAAGCGTGTTCTCTGATCATAAATGTTTGATGTTTAGAATAAGATTTGATAAGATTTTTGAAGTTTTTAATCATCCAAATGTACTTACCAGGAGGTCTTTGTTTGGAATGTCGTCGAAGAGGGAGGGAAGGATCCAGCCCTCCGTGACGAGCCAACCTCCGAGGTTCACCGCCCGAATGGGGGGCGACGGGGCGCCCCGTTTCGCGGGCCGCACCGTCCTCCCATCGGAGACTGAGAAGAGGCACGGCGAGCAGAGGAGGAGGAAGAGGAGGAGCTCAAAATGGCGCATGATGAAGACGAAGATTTTTCAGCTCTTGATGACTATCTTAGCTCAGCTTGCCAATGTATATATATACACAACGTAATGCAGCGATCGATGCATGTGCGTGTACGAACTGCGTAATTAATGGATAGACGTGCGTGCTTAGAATAAAAATGTGGACATCCAGAGTTGAATAGCTTGATGCCTACCCCATCCGTCCCAAAAAAAAAGACAAACCCTAATTTCCGTGCTCAACGTTTGACCGTCCGTCTTATTTGAAAAAATTATGAAAAAAATTAAAAAGACAAGTCACGCATAAAATATTAATCATGTTTTATCATCTAACAATAATAAAAATACGAATTATAAAAAAATTTCATATAATACGTACAGTTAAAGTTGGACACCAAAACTCAAAGGTTTTTTTTTTTAATTTACGGGAGGGAGTACGTGCGACGTTGTTGGCGTCCTGCACGCTTAATAATGAAGCTGCCTTGACGTTGAACCGCAGCCGTCGATCGATGTGCGACTGGCGGAATGGCATTGATTGGACCGTCCAACTCCGCCGGGTGGACCGT |
| 1365 | CGAGACGCTGGATTCATCCTAGATGCATAGAAAAGCGTGTTCTCTGATCATAAATGTTTGATGTTTAGAATAAGATTTGATAAGATTTTTGAAGTTTTTAATCATCCAAATGTACTTACCAGGAGGTCTTTGTTTGGAATGTCGTCGAAGAGGGAGGGAAGGATCCAGCCCTCCGTGACGAGCCAACCTCCGAGGTTCACCGCCCGAATGGGGGGCGACGGGGCGCCCCGTTTCGCGGGCCGCACCGTCCTCCCATCGGAGACTGAGAAGAGGCACGGCGAGCAGAGGAGGAGGAAGAGGAGGAGCTCAAAATGGCGCATGATGAAGACGAAGATTTTTCAGCTCTTGATGACTATCTTAGCTCAGCTTGCCAATGTATATATATACACAACGTAATGCAGCGATCGATGCATGTGCGTGTACGAACTGCGTAATTAATGGATAGACGTGCGTGCTTAGAATAAAAATGTGGACATCCAGAGTTGAATAGCTTGATGCCTACCCCATCCGTCCCAAAAAAAAAGACAAACCCTAATTTCCGTGCTCAACGTTTGACCGTCCGTCTTATTTGAAAAAATTATGAAAAAAATTAAAAAGACAAGTCACGCATAAAATATTAATCATGTTTTATCATCTAACAATAATAAAAATACGAATTATAAAAAAATTTCATATAATACGTACAGTTAAAGTTGGACACCAAAACTCAAAGGTTTTTTTTTTTTAATTTACGGAGGGGAGTACGTGCGACGTTGTTGGGCGTCCTGCACGCTTTATAAATGAAGCTGCTTGACGTTGACCGCAGCGTCGATCGATGTGCGCTGCGGAATGCAATTGATTGACCCGTACAACTCCCGCCGGGTGG |
| HJX74 | TCCTATATGCATAGAAAGCGTGTTCT*ATGTTTGATGTTTAGAATAAGATTTGATAAGATTTTTGAAGTTTTTAATCATCCAAATGTACTTACCAGGAGGTCTTTGTTTGGAATGTCGTCGAAGAGGGAGGGAAGGATCCAGCCCTCCGTGACGAGCCAACCTCCGAGGTTCACCGCCCGAATGGGGGGCGACGGGGCGCCCCGTTTCGCGGGCCGCACCGTCCTCCCATCGGAGACTGAGAAGAGGCACGGCGAGCAGAGGAGGAGGAAGAGGAGGAGCTCAAAATGGCGCATGATGAAGACGAAGATTTTTCAGCTCTTGATGACTATCTTAGCTCAGCTTGCCAATGTATATATATACACAACGTAATGCAGCGATCGATGCATGTGCGTGTACGAACTGCGTAATTAATGGATAGACGTGCGTGCTTAGAATAAAAATGTGGACATCCAGAGTTGAATAGCTTGATGCCTAC*GTGCGACGTTGTTGGCGTCCTGCACGCTTAATAATGAAGCTGCTTTGACGTTTGACCGCAGCCGTCCGATCGATGGTGCGGCTGGCCGGATGCCATTTGGTTTGACCCGTCAAACTCCGCCGGTGGCCGTCGGATGCTCATCGAACGGATATCGCATCGATCTTCAGATAATCGTCAGATTGGATAACGTATACGGATTAGTTCCGATCCAGGGGGTGTGGATTCAGCGGCGCGGGCAAATTCTATTGCGTGGGG |
| 66 | ATCCTAAATGCATAGAAAGCGTGTTCT*ATGTTTGATGTTTAGAATAAGATTTGATAAGATTTTTGAAGTTTTTAATCATCCAAATGTACTTACCAGGAGGTCTTTGTTTGGAATGTCGTCGAAGAGGGAGGGAAGGATCCAGCCCTCCGTGACGAGCCAACCTCCGAGGTTCACCGCCCGAATGGGGGGCGACGGGGCGCCCCGTTTCGCGGGCCGCACCGTCCTCCCATCGGAGACTGAGAAGAGGCACGGCGAGCAGAGGAGGAGGAAGAGGAGGAGCTCAAAATGGCGCATGATGAAGACGAAGATTTTTCAGCTCTTGATGACTATCTTAGCTCAGCTTGCCAATGTATATATATACACAACGTAATGCAGCGATCGATGCATGTGCGTGTACGAACTGCGTAATTAATGGATAGACGTGCGTGCTTAGAATAAAAATGTGGACATCCAGAGTTGAATAGCTTGATGCCTAC*GTGCGACGTTGTTGGCGTCCTGCACGCTTAATAATGAAGCTGCTTTGACGTTTGACCGCAGCCGTCCGATCGATGGTGCGGCTGGCCGGATGCCATTTGGTTTGACCCGTCAAACTCCGCCGGTGGCCGTCGGATGCTCATCGAACGGATATCGCATCGATCTTCAGATAATCGTCAGATTGGATAACGTATACGGATTAGTTCCGATCCAGGGGTGTGGATTCAGCGGCGCGGGCAAATTCTATTGGCTGTGGTTGAGCCTATCCTAA |
| 550 | AGCTGGACTCATCCTAGATGCATAGAAAGCGTGTTCT*ATGTTTGATGTTTAGAATAAGATTTGATAAGATTTTTGAAGTTTTTAATCATCCAAATGTACTTACCAGGAGGTCTTTGTTTGGAATGTCGTCGAAGAGGGAGGGAAGGATCCAGCCCTCCGTGACGAGCCAACCTCCGAGGTTCACCGCCCGAATGGGGGGCGACGGGGCGCCCCGTTTCGCGGGCCGCACCGTCCTCCCATCGGAGACTGAGAAGAGGCACGGCGAGCAGAGGAGGAGGAAGAGGAGGAGCTCAAAATGGCGCATGATGAAGACGAAGATTTTTCAGCTCTTGATGACTATCTTAGCTCAGCTTGCCAATGTATATATATACACAACGTAATGCAGCGATCGATGCATGTGCGTGTACGAACTGCGTAATTAATGGATAGACGTGCGTGCTTAGAATAAAAATGTGGACATCCAGAGTTGAATAGCTTGATGCCTAC*GTGCGACGTTGTTGGCGTCCTGCACGCTTAATAATGAAGCTGCTTTGACGTTTGACCGCAGCCGTCCGATCGATGGTGCGGCTGGCCGGATGCCATTTGGTTTGACCCGTCAAACTCCGCCGGTGGCCGTCGGATGCTCATCGAACGGATATCGCATCGATCTTCAGATAATCGTCAGATTGGATAACGTATACGGATTAGTTCCGATCCAGGGGTGTGGATTCAGCGGCGCGGGCAAATTCTATTGTC |
| 578 | GGTTCATCCTAAATGCATAGAAAGCGTGTTCT*ATGTTTGATGTTTAGAATAAGATTTGATAAGATTTTTGAAGTTTTTAATCATCCAAATGTACTTACCAGGAGGTCTTTGTTTGGAATGTCGTCGAAGAGGGAGGGAAGGATCCAGCCCTCCGTGACGAGCCAACCTCCGAGGTTCACCGCCCGAATGGGGGGCGACGGGGCGCCCCGTTTCGCGGGCCGCACCGTCCTCCCATCGGAGACTGAGAAGAGGCACGGCGAGCAGAGGAGGAGGAAGAGGAGGAGCTCAAAATGGCGCATGATGAAGACGAAGATTTTTCAGCTCTTGATGACTATCTTAGCTCAGCTTGCCAATGTATATATATACACAACGTAATGCAGCGATCGATGCATGTGCGTGTACGAACTGCGTAATTAATGGATAGACGTGCGTGCTTAGAATAAAAATGTGGACATCCAGAGTTGAATAGCTTGATGCCTAC*GTGCGACGTTGTTGGCGTCCTGCACGCTTAATAATGAAGCTGCTTTGACGTTTGACCGCAGCCGTCCGATCGATGGTGCGGCTGGCCGGATGCCATTTGGTTTGACCCGTCAAACTCCGCCGGTGGCCGTCGGATGCTCATCGAACGGATATCGCATCGATCTTCAGATAATCGTCAGATTGGATAACGTATACGGATTAGTTCCGATCCAGGGGTGTGGATTCAGCGGCGCGGGCAATTCTATGGGGGGGGGAGGCATAAAAAA |
| 612 | GTTCATCCTAGATGCATAGAAAGCGTGTTCT*ATGTTTGATGTTTAGAATAAGATTTGATAAGATTTTTGAAGTTTTTAATCATCCAAATGTACTTACCAGGAGGTCTTTGTTTGGAATGTCGTCGAAGAGGGAGGGAAGGATCCAGCCCTCCGTGACGAGCCAACCTCCGAGGTTCACCGCCCGAATGGGGGGCGACGGGGCGCCCCGTTTCGCGGGCCGCACCGTCCTCCCATCGGAGACTGAGAAGAGGCACGGCGAGCAGAGGAGGAGGAAGAGGAGGAGCTCAAAATGGCGCATGATGAAGACGAAGATTTTTCAGCTCTTGATGACTATCTTAGCTCAGCTTGCCAATGTATATATATACACAACGTAATGCAGCGATCGATGCATGTGCGTGTACGAACTGCGTAATTAATGGATAGACGTGCGTGCTTAGAATAAAAATGTGGACATCCAGAGTTGAATAGCTTGATGCCTAC*GTGCGACGTTGTTGGCGTCCTGCACGCTTAATAATGAAGCTGCTTTGACGTTTGACCGCAGCCGTCCGATCGATGGTGCGGCTGGCCGGATGCCATTTGGTTTGACCCGTCAAACTCCGCCGGTGGCCGTCGGATGCTCATCGAACGGATATCGCATCGATCTTCAGATAATCGTCAGATTGGATAACGTATACGGATTAGTTCCGATCCAGGGGGTGTGGATTCAGCGGCGCGGGCAAATTCTATTGCCTGG |
| 628 | TCCTAGATGCATAGAAAGCGTGTTCT*ATGTTTGATGTTTAGAATAAGATTTGATAAGATTTTTGAAGTTTTTAATCATCCAAATGTACTTACCAGGAGGTCTTTGTTTGGAATGTCGTCGAAGAGGGAGGGAAGGATCCAGCCCTCCGTGACGAGCCAACCTCCGAGGTTCACCGCCCGAATGGGGGGCGACGGGGCGCCCCGTTTCGCGGGCCGCACCGTCCTCCCATCGGAGACTGAGAAGAGGCACGGCGAGCAGAGGAGGAGGAAGAGGAGGAGCTCAAAATGGCGCATGATGAAGACGAAGATTTTTCAGCTCTTGATGACTATCTTAGCTCAGCTTGCCAATGTATATATATACACAACGTAATGCAGCGATCGATGCATGTGCGTGTACGAACTGCGTAATTAATGGATAGACGTGCGTGCTTAGAATAAAAATGTGGACATCCAGAGTTGAATAGCTTGATGCCTAC*GTGCGACGTTGTTGGCGTCCTGCACGCTTAATAATGAAGCTGCTTTGACGTTTGACCGCAGCCGTCCGATCGATGGTGCGGCTGGCCGGATGCCATTTGGTTTGACCCGTCAAACTCCGCCGGTGGCCGTCGGATGCTCATCGAACGGATATCGCATCGATCTTCAGATAATCGTCAGATTGGATAACGTATACGGATTAGTTCCGATCCAGGGGGTGTGGATTCAGCGGCGCGGGGCAAATTCTATTGGCTG |
| 1154 | TGCATAGAAAGCGTGTTCT*ATGTTTGATGTTTAGAATAAGATTTGATAAGATTTTTGAAGTTTTTAATCATCCAAATGTACTTACCAGGAGGTCTTTGTTTGGAATGTCGTCGAAGAGGGAGGGAAGGATCCAGCCCTCCGTGACGAGCCAACCTCCGAGGTTCACCGCCCGAATGGGGGGCGACGGGGCGCCCCGTTTCGCGGGCCGCACCGTCCTCCCATCGGAGACTGAGAAGAGGCACGGCGAGCAGAGGAGGAGGAAGAGGAGGAGCTCAAAATGGCGCATGATGAAGACGAAGATTTTTCAGCTCTTGATGACTATCTTAGCTCAGCTTGCCAATGTATATATATACACAACGTAATGCAGCGATCGATGCATGTGCGTGTACGAACTGCGTAATTAATGGATAGACGTGCGTGCTTAGAATAAAAATGTGGACATCCAGAGTTGAATAGCTTGATGCCTAC*GTGCGACGTTGTTGGCGTCCTGCACGCTTAATAATGAAGCTGCTTTGACGTTTGACCGCAGCCGTCCGATCGATGGTGCGGCTGGCCGGATGCCATTTGGTTTGACCCGTCAAACTCCGCCGGTGGCCGTCGGATGCTCATCGAACGGATATCGCATCGATCTTCAGATAATCGTCAGATTGGATAACGTATACGGATTAGTTCCGATCCAGGGGTGTGGATTCAGCGGCGCGGGCAAATTCTATGTGGGGGGGGGCTTTAAAAA |
| 484 | GGTTCATCCTAAATGCATAGAAaAGCGTGTTCT*ATGTTTGATGTTTAGAATAAGATTTGATAAGATTTTTGAAGTTTTTAATCATCCAAATGTACTTACCAGGAGGTCTTTGTTTGGAATGTCGTCGAAGAGGGAGGGAAGGATCCAGCCCTCCGTGACGAGCCAACCTCCGAGGTTCACCGCCCGAATGGGGGGCGACGGGGCGCCCCGTTTCGCGGGCCGCACCGTCCTCCCATCGGAGACTGAGAAGAGGCACGGCGAGCAGAGGAGGAGGAAGAGGAGGAGCTCAAAATGGCGCATGATGAAGACGAAGATTTTTCAGCTCTTGATGACTATCTTAGCTCAGCTTGCCAATGTATATATATACACAACGTAATGCAGCGATCGATGCATGTGCGTGTACGAACTGCGTAATTAATGGATAGACGTGCGTGCTTAGAATAAAAATGTGGACATCCAGAGTTGAATAGCTTGATGCCTAC*GTGCGACGTTGTTGGCGTCCTGCACGCTTAATAATGAAGCTGCTTTGACGTTTGACCGCAGCCGTCCGATCGATGGTGCGGCTGGCCGGATGCCATTTGGTTTGACCCGTCAAACTCCGCCGGTGGCCGTCGGATGCTCATCGAACGGATATCGCATCGATCTTCAGATAATCGTCAGATTGGATAACGTATACGGATTAGTTCCGATCCAGGGGGTGTGGATTCAGCGGCGCGGGCAAATTCTATTGGTCCTGCGGGTGGAGGTCTCTCA |
| 86 | CCTAAATGCATAGAAAGCGTGTTCT*ATGTTTGATGTTTAGAATAAGATTTGATAAGATTTTTGAAGTTTTTAATCATCCAAATGTACTTACCAGGAGGTCTTTGTTTGGAATGTCGTCGAAGAGGGAGGGAAGGATCCAGCCCTCCGTGACGAGCCAACCTCCGAGGTTCACCGCCCGAATGGGGGGCGACGGGGCGCCCCGTTTCGCGGGCCGCACCGTCCTCCCATCGGAGACTGAGAAGAGGCACGGCGAGCAGAGGAGGAGGAAGAGGAGGAGCTCAAAATGGCGCATGATGAAGACGAAGATTTTTCAGCTCTTGATGACTATCTTAGCTCAGCTTGCCAATGTATATATATACACAACGTAATGCAGCGATCGATGCATGTGCGTGTACGAACTGCGTAATTAATGGATAGACGTGCGTGCTTAGAATAAAAATGTGGACATCCAGAGTTGAATAGCTTGATGCCTAC*GTGCGACGTTGTTGGCGTCCTGCACGCTTAATAATGAAGCTGCTTTGACGTTTGACCGCAGCCGTCCGATCGATGGTGCGGCTGGCCGGATGCCATTTGGTTTGACCCGTCAAACTCCGCCGGTGGCCGTCGGATGCTCATCGAACGGATATCGCATCGATCTTCAGATAATCGTCAGATTGGATAACGTATACGGATTAGTTCCGATCCAGGGGTGTGGATTCAGCGGCGCGGGCAAATTCTATTGGTGCGTGTGATGACCTTCCCA |
| 1400 | AAGTAAGCTGGGTTCATCCTATATGCATAGAAAGCGTGTTCT*ATGTTTGATGTTTAGAATAAGATTTGATAAGATTTTTGAAGTTTTTAATCATCCAAATGTACTTACCAGGAGGTCTTTGTTTGGAATGTCGTCGAAGAGGGAGGGAAGGATCCAGCCCTCCGTGACGAGCCAACCTCCGAGGTTCACCGCCCGAATGGGGGGCGACGGGGCGCCCCGTTTCGCGGGCCGCACCGTCCTCCCATCGGAGACTGAGAAGAGGCACGGCGAGCAGAGGAGGAGGAAGAGGAGGAGCTCAAAATGGCGCATGATGAAGACGAAGATTTTTCAGCTCTTGATGACTATCTTAGCTCAGCTTGCCAATGTATATATATACACAACGTAATGCAGCGATCGATGCATGTGCGTGTACGAACTGCGTAATTAATGGATAGACGTGCGTGCTTAGAATAAAAATGTGGACATCCAGAGTTGAATAGCTTGATGCCTAC*GTGCGACGTTGTTGGCGTCCTGCACGCTTAATAATGAAGCTGCTTTGACGTTTGACCGCAGCCGTCCGATCGATGGTGCGGCTGGCCGGATGCCATTTGGTTTGACCCGTCAAACTCCGCCGGTGGCCGTCGGATGCTCATCGAACGGATATCGCATCGATCTTCAGATAATCGTCAGATTGGATAACGTATACGGATTAGTTCCGATCCAGGGGTGTGGATTCAGCGGCGCGGGCAAATTCTATTGGCTCGTGGGGAGCTTTCAA |

Note: * insertion sites.
